# Supplementary material for: A muscle-epidermis-glia signaling axis sustains synaptic specificity during allometric growth in Caenorhabditis elegans
Source: eLife. 2020 Apr 7;9:e55890. doi: 10.7554/eLife.55890 (PMC7164957; doi:10.7554/eLife.55890)
Supplement: Supplementary file 1. — Proteins upregulated (>1.2 fold) or downregulated (<0.8 fold) are listed in the spreadsheet. Note that mig-17(ola226) was isolated from forward genetic screen, which would introduce other background mutations. Further analyses are required to determine if the protein level changes are due specifically to the mig-17(ola226) mutation. [file elife-55890-supp1.pdf]

Table S1 the results of MS

Accession	Gene names	N2 repeat 1	N2 repeat 2	N2 repeat 3	average	mig-17(ola226) repeat 1	mig-17(ola226) repeat 2	mig-17(ola226) repeat 3	average	fold change
Q20877	CELE_F56D3.1 F56D3.1	0.81	0.56	0.00	0.46	0.19	0.00	0.00	0.06	0.14
P30629	ZK637.2	1.00	1.00	0.36	0.79	0.00	0.00	0.50	0.17	0.21
Q20049	idhg-1 CELE_F35G12.2 F35G12.2	0.42	1.00	0.00	0.47	0.41	0.00	0.00	0.14	0.29
Q21742	CELE_R05F9.6 R05F9.6	0.33	0.00	1.00	0.44	0.43	0.00	0.00	0.14	0.32
Q9XW20	CELE_Y18D10A.11 Y18D10A.11	0.35	0.00	1.00	0.45	0.44	0.00	0.00	0.15	0.32
Q09482	C18H9.3/C18H9.2	0.00	1.00	0.33	0.44	0.00	0.00	0.44	0.15	0.33
Q2EEM8	ttr-45 CELE_JC8.14 JC8.14	0.27	0.47	0.56	0.43	0.48	0.00	0.00	0.16	0.37
Q20223	lbp-1 F40F4.3	0.00	0.33	0.57	0.30	0.00	0.38	0.00	0.13	0.41
O44144	perm-4 C44B12.5 CELE_C44B12.5	0.34	0.50	1.00	0.61	0.38	0.39	0.00	0.26	0.42
Q22015	CELE_R31.2 R31.2	0.00	0.00	0.51	0.17	0.00	0.00	0.21	0.07	0.42
Q9GUF2	acp-6 CELE_Y73B6BL.24 Y73B6BL.24	0.00	0.34	0.50	0.28	0.00	0.38	0.00	0.13	0.46
G5EGA7	ras-2 Ras2 CELE_F17C8.4 F17C8.4	0.00	0.25	0.00	0.08	0.00	0.11	0.00	0.04	0.46
O45006	CELE_W03D8.9 W03D8.9	1.00	0.00	0.18	0.39	0.00	0.00	0.55	0.18	0.46
P34654	ZK632.9	0.00	0.51	0.00	0.17	0.00	0.24	0.00	0.08	0.47
Q9Y0V6	tin-10 tim-10 Y66D12A.22	0.00	0.00	0.68	0.23	0.00	0.00	0.33	0.11	0.48
Q9XWU9	CELE_Y37D8A.19 Y37D8A.19	0.50	0.00	0.41	0.30	0.20	0.00	0.24	0.15	0.48
O76618	cars-1 CELE_Y23H5A.7 Y23H5A.7	0.00	0.00	0.49	0.16	0.00	0.00	0.24	0.08	0.49
Q9UB28	let-805 CELE_H19M22.2 H19M22.2	0.28	0.00	0.59	0.29	0.45	0.00	0.00	0.15	0.52
Q966D6	mct-2 mct-1 C01B4.9 CELE_C01B4.9 CELE_Y19D10A.12 Y19D10A.12	0.00	1.00	0.00	0.33	0.00	0.53	0.00	0.18	0.53
P91340	CELE_F55F8.2 F55F8.2	0.00	0.00	0.55	0.18	0.00	0.00	0.30	0.10	0.53
Q22782	rab-6.2 T25G12.4	0.00	0.64	0.00	0.21	0.00	0.36	0.00	0.12	0.57
P91280	CELE_F27C1.6 F27C1.6	0.00	0.00	0.55	0.18	0.00	0.00	0.32	0.11	0.57
Q18032	C15H9.9 CELE_C15H9.9	0.28	1.00	0.31	0.53	0.47	0.00	0.44	0.30	0.57
O44954	sdha-2 C34B2.7 CELE_C34B2.7	0.50	0.00	0.00	0.17	0.29	0.00	0.00	0.10	0.59
O44572	tni-4 W03F8.1	0.00	0.00	0.62	0.21	0.00	0.00	0.38	0.13	0.60
Q20277	fipr-21 CELE_F41E7.5 F41E7.5	0.35	0.51	0.34	0.40	0.37	0.00	0.37	0.25	0.62
G5EFB5	cpn-1 CELE_F43G9.9 F43G9.9	0.50	0.46	0.31	0.42	0.25	0.15	0.40	0.27	0.63
Q17435	pfd-4 tag-317 B0035.4	0.55	0.32	0.00	0.29	0.00	0.56	0.00	0.19	0.64
Q9XWK2	CELE_Y54E5A.5 Y54E5A.5	0.46	0.48	0.78	0.57	0.37	0.35	0.40	0.37	0.65
Q21322	rnp-2 CELE_K08D10.4 K08D10.4	0.00	0.30	0.00	0.10	0.00	0.20	0.00	0.07	0.65
O45430	mccc-1 CELE_F32B6.2 F32B6.2	0.31	0.31	0.00	0.21	0.00	0.42	0.00	0.14	0.67
O44145	perm-2 C44B12.1 CELE_C44B12.1	0.28	0.16	0.65	0.36	0.34	0.41	0.00	0.25	0.69
Q9GZH3	T22D1.3	0.15	1.00	0.41	0.52	0.58	0.00	0.52	0.37	0.71
Q9XWT3	ule-5 CELE_Y62H9A.6 Y62H9A.6	0.36	0.55	0.00	0.30	0.39	0.26	0.00	0.22	0.71
Q94148	rab-10 T23H2.5	0.00	0.58	0.00	0.19	0.00	0.42	0.00	0.14	0.72
O17891	CELE_F55B11.2 F55B11.2	0.00	0.00	0.45	0.15	0.00	0.00	0.33	0.11	0.72
Q23028	CELE_R09F10.5 R09F10.5	0.60	0.46	0.46	0.50	0.40	0.33	0.37	0.37	0.73
Q9XXK7	tram-1 C24F3.1 CELE_C24F3.1	0.00	0.45	0.00	0.15	0.00	0.34	0.00	0.11	0.76
P41847	T20B12.7	0.53	0.44	0.00	0.32	0.35	0.39	0.00	0.25	0.77
P34640	ZK512.2	0.00	0.57	0.00	0.19	0.00	0.43	0.00	0.14	0.77
G5EET8	pud-1.2 pud-1.1 CELE_F15E11.13 CELE_Y19D10B.7 F15E11.13 Y19D10B.7	0.36	0.35	0.37	0.36	0.27	0.29	0.26	0.28	0.77
Q9BKU8	moag-4 CELE_Y37E3.4 Y37E3.4	0.00	0.00	0.43	0.14	0.00	0.00	0.34	0.11	0.78
Q94053	msp-78 T13F2.11	0.55	0.57	0.31	0.48	0.40	0.36	0.36	0.37	0.78
Q21774	adsl-1 R06C7.5	0.41	0.00	0.00	0.14	0.32	0.00	0.00	0.11	0.79
Q9TYV5	nol-1 CELE_W07E6.1 W07E6.1	0.00	0.48	0.45	0.31	0.00	0.73	0.00	0.24	0.79
Q9N4I3	CELE_Y71F9AL.9 Y71F9AL.9	0.41	0.38	0.32	0.37	0.44	0.50	0.39	0.44	1.20
Q9NAR3	col-124 C24F3.6 CELE_C24F3.6	0.31	0.34	0.35	0.33	0.26	0.49	0.46	0.40	1.20
Q9N4N4	swsn-6 CELE_ZK616.4 ZK616.4	0.36	0.00	0.00	0.12	0.44	0.00	0.00	0.15	1.20

Q9TZ33	ucr-2.3 CELE_T24C4.1 T24C4.1	0.00	0.32	0.38	0.23	0.00	0.43	0.42	0.28	1.20
P19625	mlc-1 C36E6.3	0.00	0.00	0.45	0.15	0.00	0.00	0.55	0.18	1.21
Q9XVS1	tag-72 C25A1.3	0.00	0.00	0.45	0.15	0.00	0.00	0.55	0.18	1.21
Q9XXE2	CELE_Y44A6D.2 Y44A6D.2	0.00	0.37	0.00	0.12	0.00	0.44	0.00	0.15	1.21
P34685	cap-1 D2024.6	0.29	0.00	0.37	0.22	0.45	0.00	0.35	0.27	1.21
O62053	C08F11.11	0.33	0.34	0.32	0.33	0.42	0.39	0.40	0.40	1.21
H2KZV8	mlp-1 CELE_T04C9.4 T04C9.4	0.35	0.34	0.33	0.34	0.44	0.39	0.41	0.41	1.21
Q20588	F49C12.11	0.00	0.29	0.00	0.10	0.00	0.36	0.00	0.12	1.22
O01805	acbp-1 C44E4.6	0.32	0.39	0.34	0.35	0.44	0.42	0.43	0.43	1.22
Q27481	uba-1 C47E12.5 CELE_C47E12.5	0.35	0.33	0.39	0.35	0.40	0.47	0.42	0.43	1.22
P91303	vha-10 F46F11.5	0.34	0.35	0.34	0.34	0.42	0.41	0.42	0.41	1.22
Q93568	CELE_F25H2.4 F25H2.4	0.00	0.00	0.45	0.15	0.00	0.00	0.55	0.18	1.22
Q9N2W7	Y94H6A.8	0.00	0.00	0.34	0.11	0.00	0.00	0.41	0.14	1.22
Q95PZ1	CELE_Y67H2A.5 Y67H2A.5	0.31	0.39	0.38	0.36	0.46	0.43	0.43	0.44	1.22
Q21962	CELE_R12C12.1 R12C12.1	0.38	0.26	0.00	0.21	0.39	0.39	0.00	0.26	1.22
Q9NLD1	hrp-2 CELE_F58D5.1 F58D5.1	0.33	0.32	0.38	0.34	0.43	0.43	0.40	0.42	1.22
Q95017	ubc-9 F29B9.6	0.00	0.32	0.00	0.11	0.00	0.39	0.00	0.13	1.23
Q86NE1	asp-2 CELE_T18H9.2 T18H9.2	0.32	0.36	0.35	0.34	0.42	0.42	0.42	0.42	1.23
P34339	egl-45 eif-3.A C27D11.1	0.28	0.32	0.40	0.33	0.40	0.41	0.41	0.41	1.23
Q95YB2	acdh-9 CELE_F28A10.6 F28A10.6	0.00	0.47	0.29	0.25	0.00	0.47	0.46	0.31	1.23
Q94010	CELE_T08G11.1 T08G11.1	0.45	0.53	0.37	0.45	0.55	0.47	0.63	0.55	1.23
O45569	nep-17 CELE_F54F11.2 F54F11.2	0.00	0.00	0.34	0.11	0.00	0.00	0.41	0.14	1.23
Q17698	C06A8.3 CELE_C06A8.3	0.31	0.32	0.32	0.32	0.39	0.38	0.39	0.39	1.23
G5ECW7	dpt-1 CELE_F02E9.9 F02E9.9	0.00	0.00	0.41	0.14	0.00	0.00	0.50	0.17	1.23
P18835	col-19 ZK1193.1	0.29	0.00	0.00	0.10	0.36	0.00	0.00	0.12	1.24
Q19766	tomm-20 F23H12.2	0.30	0.29	0.34	0.31	0.38	0.43	0.35	0.38	1.24
O17328	mct-6 C10E2.6 CELE_C10E2.6	0.37	0.37	0.00	0.24	0.51	0.40	0.00	0.30	1.24
P55956	asp-3 H22K11.1	0.32	0.35	0.34	0.34	0.42	0.42	0.42	0.42	1.24
P91027	chdp-1 C10G11.7 CELE_C10G11.7	0.30	0.42	0.31	0.34	0.46	0.33	0.50	0.43	1.24
Q09936	C53C9.2	0.32	0.32	0.33	0.32	0.41	0.38	0.41	0.40	1.24
Q22352	CELE_T08H10.1 T08H10.1	0.33	0.36	0.33	0.34	0.46	0.37	0.44	0.42	1.24
P31161	sod-2 sdm-1 F10D11.1	0.32	0.31	0.45	0.36	0.44	0.46	0.45	0.45	1.24
Q9N408	ddp-1 tim-8 Y39A3CR.4	0.43	0.27	0.44	0.38	0.57	0.43	0.41	0.47	1.24
O02115	pcn-1 W03D2.4	0.37	0.32	0.24	0.31	0.34	0.40	0.42	0.39	1.25
G3MU83	math-33 CELE_H19N07.2 H19N07.2	0.32	0.32	0.37	0.33	0.43	0.42	0.40	0.42	1.25
P55955	ttr-16 Y5F2A.1	0.54	0.28	0.30	0.37	0.52	0.44	0.43	0.46	1.25
Q9U296	men-1 CELE_Y48B6A.12 Y48B6A.12	0.00	0.00	0.38	0.13	0.00	0.00	0.47	0.16	1.25
Q20774	dnj-13 CELE_F54D5.8 F54D5.8	0.00	0.00	0.34	0.11	0.00	0.00	0.43	0.14	1.25
P91306	cey-2 CELE_F46F11.2 F46F11.2	0.33	0.35	0.32	0.33	0.41	0.43	0.41	0.42	1.25
Q19826	rpb-8 F26F4.11	0.35	0.00	0.00	0.12	0.44	0.00	0.00	0.15	1.25
Q9XXS2	set-22 CELE_Y32F6A.1 Y32F6A.1	0.00	0.00	0.35	0.12	0.00	0.00	0.44	0.15	1.25
O02323	lbp-7 T22G5.2	0.32	0.00	0.31	0.21	0.43	0.00	0.36	0.26	1.26
Q17820	aos-1 C08B6.9	0.00	0.33	0.00	0.11	0.00	0.41	0.00	0.14	1.26
O44955	C34B2.8 CELE_C34B2.8	0.00	0.32	0.00	0.11	0.00	0.40	0.00	0.13	1.26
Q21217	gta-1 K04D7.3	0.34	0.31	0.33	0.33	0.41	0.40	0.42	0.41	1.26
O17607	ruvb-1 C27H6.2	0.30	0.36	0.34	0.33	0.43	0.40	0.42	0.42	1.26
Q27535	ZC434.8	0.32	0.34	0.35	0.34	0.40	0.44	0.43	0.42	1.26
Q19842	pcca-1 F27D9.5	0.33	0.42	0.28	0.35	0.47	0.36	0.47	0.43	1.26
Q20107	fkb-1 CELE_F36H1.1 F36H1.1	0.35	0.00	0.00	0.12	0.44	0.00	0.00	0.15	1.26
Q20310	CELE_F42A10.5 F42A10.5	0.39	0.00	0.29	0.22	0.42	0.00	0.43	0.28	1.26

Q22235	enpl-1 T05E11.3	0.31	0.36	0.34	0.34	0.41	0.42	0.45	0.43	1.26
P34388	mrps-9 F09G8.3	0.00	0.04	0.00	0.01	0.00	0.05	0.00	0.02	1.26
Q93714	idha-1 F43G9.1	0.33	0.34	0.35	0.34	0.44	0.42	0.43	0.43	1.26
O62388	CELE_W01D2.1 W01D2.1	0.34	0.32	0.33	0.33	0.41	0.42	0.41	0.41	1.27
Q2XN18	spp-14 CELE_K09F5.3 K09F5.3	0.35	0.31	0.31	0.32	0.35	0.43	0.44	0.41	1.27
Q21763	CELE_R05H5.3 R05H5.3	0.35	0.28	0.38	0.34	0.44	0.45	0.40	0.43	1.27
Q21000	myo-5 CELE_F58G4.1 F58G4.1	0.35	0.30	0.51	0.39	0.54	0.47	0.46	0.49	1.27
H2FLH2	unc-22 CELE_ZK617.1 ZK617.1	0.25	0.37	0.40	0.34	0.43	0.41	0.45	0.43	1.27
Q9XU97	CELE_F44E5.1 F44E5.1	0.37	0.32	0.28	0.32	0.36	0.43	0.45	0.41	1.27
Q18599	acer-1 C44B7.10 CELE_C44B7.10	0.33	0.29	0.33	0.32	0.41	0.42	0.38	0.40	1.27
Q22037	hrp-1 rbp-1 F42A6.7	0.34	0.35	0.36	0.35	0.43	0.49	0.42	0.45	1.27
O45924	Y39E4A.3	0.32	0.33	0.32	0.32	0.43	0.43	0.37	0.41	1.27
Q22781	acdh-7 CELE_T25G12.5 T25G12.5	0.36	0.36	0.29	0.34	0.41	0.43	0.45	0.43	1.27
Q23445	ZK180.4	0.33	0.00	0.00	0.11	0.42	0.00	0.00	0.14	1.27
Q19264	F09E5.3	0.33	0.00	0.34	0.22	0.44	0.00	0.42	0.29	1.28
Q09359	ZK1307.1	0.35	0.34	0.32	0.34	0.43	0.42	0.44	0.43	1.28
Q9XXU9	vha-11 Y38F2AL.3	0.39	0.34	0.32	0.35	0.46	0.43	0.46	0.45	1.28
P34462	vha-14 F55H2.2	0.31	0.47	0.30	0.36	0.42	0.53	0.43	0.46	1.28
P52009	cyn-1 cyp-1 Y49A3A.5	0.38	0.30	0.30	0.33	0.42	0.43	0.40	0.42	1.28
G5ED41	cand-1 Y102A5A.1	0.00	0.00	0.30	0.10	0.00	0.00	0.38	0.13	1.28
O01602	nuo-2 CELE_T10E9.7 T10E9.7	0.00	0.00	0.32	0.11	0.00	0.00	0.42	0.14	1.28
Q23280	daf-41 p23 ZC395.10	0.28	0.51	0.28	0.36	0.46	0.45	0.46	0.46	1.28
Q10453	his-71 F45E1.6	0.28	0.52	0.29	0.36	0.48	0.48	0.44	0.47	1.28
O62289	ttr-51 CELE_JC8.8 JC8.8	0.34	0.31	0.33	0.33	0.41	0.43	0.42	0.42	1.28
P53596	sucl-1 C05G5.4	0.36	0.32	0.34	0.34	0.41	0.47	0.43	0.44	1.28
Q09508	sdha-1 C03G5.1/D2021.3	0.35	0.41	0.34	0.37	0.52	0.44	0.46	0.47	1.28
Q20239	aagr-3 CELE_F40F9.6 F40F9.6	0.00	0.44	0.00	0.15	0.00	0.56	0.00	0.19	1.29
Q93353	idhb-1 C37E2.1	0.35	0.36	0.31	0.34	0.43	0.47	0.41	0.44	1.29
Q20922	col-159 CELE_F57B1.3 F57B1.3	0.00	0.29	0.00	0.10	0.00	0.38	0.00	0.13	1.29
P90901	ifa-1 F38B2.1	0.35	0.36	0.33	0.34	0.52	0.36	0.45	0.44	1.29
Q9U2S6	cyn-13 CELE_Y116A8C.34 Y116A8C.34	0.33	0.38	0.41	0.37	0.42	0.44	0.59	0.48	1.29
Q23098	nuo-6 CELE_W01A8.4 W01A8.4	0.26	0.27	0.48	0.34	0.47	0.43	0.41	0.44	1.29
Q03565	baf-1 B0464.7	0.32	0.34	0.32	0.32	0.44	0.42	0.40	0.42	1.29
O61793	CELE_R12E2.13 R12E2.13	0.39	0.00	0.29	0.23	0.39	0.00	0.49	0.30	1.29
O01615	T19H12.2	0.49	0.26	0.33	0.36	0.30	0.45	0.67	0.47	1.30
O01532	asp-5 CELE_F21F8.3 F21F8.3	0.31	0.34	0.33	0.32	0.42	0.44	0.40	0.42	1.30
Q09EE7	nsf-1 CELE_H15N14.2 H15N14.2	0.34	0.33	0.36	0.34	0.44	0.46	0.43	0.44	1.30
Q27245	lap-2 W07G4.4	0.34	0.31	0.34	0.33	0.44	0.44	0.41	0.43	1.30
Q9TZL8	pfk-1 Y71H10A.1	0.00	0.36	0.34	0.23	0.00	0.44	0.47	0.30	1.30
H2L2E8	tba-1 CELE_F26E4.8 F26E4.8	0.31	0.00	0.00	0.10	0.40	0.00	0.00	0.13	1.30
Q9TZS5	cct-7 CELE_T10B5.5 T10B5.5	0.62	0.27	0.26	0.38	0.57	0.47	0.46	0.50	1.30
Q19832	msp-3 F26G1.7	0.35	0.00	0.34	0.23	0.44	0.00	0.46	0.30	1.30
Q9N384	lec-6 CELE_Y55B1AR.1 Y55B1AR.1	0.32	0.38	0.36	0.35	0.46	0.46	0.46	0.46	1.30
O17641	col-178 C34F6.2 CELE_C34F6.2	0.00	0.29	0.30	0.20	0.00	0.45	0.31	0.25	1.30
G4SI07	gei-15 CELE_M03A8.4 M03A8.4	0.00	0.38	0.00	0.13	0.00	0.50	0.00	0.17	1.30
Q20964	lys-4 CELE_F58B3.1 F58B3.1	0.31	0.33	0.29	0.31	0.42	0.39	0.41	0.41	1.30
Q3LFN1	lbp-9 CELE_Y40B10A.1 Y40B10A.1	0.31	0.32	0.31	0.31	0.41	0.40	0.43	0.41	1.31
Q21230	K04G2.1	0.36	0.32	0.30	0.33	0.41	0.43	0.44	0.43	1.31
P30642	eif-3.D R08D7.3	0.30	0.39	0.29	0.33	0.43	0.42	0.42	0.43	1.31
O44727	cpn-4 CELE_F49D11.8 F49D11.8	0.33	0.35	0.32	0.33	0.43	0.45	0.44	0.44	1.31

Q17512	B0491.5 CELE_B0491.5	0.40	0.29	0.49	0.39	0.51	0.47	0.57	0.51	1.31
Q95XR0	CELE_Y39G10AR.8 Y39G10AR.8	0.37	0.28	0.35	0.33	0.49	0.45	0.38	0.44	1.31
O44512	isp-1 CELE_F42G8.12 F42G8.12	0.32	0.33	0.34	0.33	0.43	0.43	0.45	0.44	1.31
Q9U2Q8	fkb-2 CELE_Y18D10A.19 Y18D10A.19	0.33	0.32	0.32	0.32	0.42	0.41	0.43	0.42	1.32
P34697	sod-1 C15F1.7	0.33	0.32	0.32	0.32	0.43	0.43	0.42	0.42	1.32
G5EDD4	tba-4 CELE_F44F4.11 F44F4.11	0.33	0.32	0.28	0.31	0.41	0.42	0.40	0.41	1.32
P19974	cyc-2.1 E04A4.7	0.33	0.34	0.34	0.34	0.45	0.44	0.44	0.44	1.32
Q9BL34	CELE_Y71H2AM.5 Y71H2AM.5	0.32	0.31	0.33	0.32	0.42	0.42	0.42	0.42	1.32
Q18231	rps-30 C26F1.4 CELE_C26F1.4	0.33	0.32	0.32	0.32	0.39	0.39	0.49	0.42	1.32
G5EBH7	calu-1 CELE_M03F4.7 M03F4.7	0.37	0.32	0.38	0.35	0.43	0.53	0.44	0.47	1.32
Q21930	rpl-28 R11D1.8	0.33	0.32	0.33	0.33	0.43	0.43	0.44	0.43	1.32
Q20363	sip-1 F43D9.4	0.32	0.34	0.32	0.33	0.43	0.44	0.43	0.43	1.32
Q95XT5	trap-1 CELE_Y71F9AM.6 Y71F9AM.6	0.32	0.33	0.32	0.32	0.42	0.43	0.42	0.43	1.32
Q9XWI6	eif-3.B Y54E2A.11	0.32	0.34	0.32	0.33	0.42	0.41	0.46	0.43	1.32
Q18211	ran-3 C26D10.1	0.41	0.33	0.28	0.34	0.46	0.41	0.48	0.45	1.32
P90983	rps-29 B0412.4 CELE_B0412.4	0.34	0.27	0.27	0.29	0.39	0.39	0.39	0.39	1.33
Q10020	T28D9.1	0.31	0.36	0.31	0.33	0.39	0.46	0.46	0.44	1.33
Q9U329	CELE_W09C5.8 W09C5.8	0.33	0.33	0.34	0.33	0.45	0.44	0.42	0.44	1.33
P34346	let-754 C29E4.8	0.32	0.32	0.33	0.32	0.44	0.42	0.42	0.43	1.33
P52011	cyn-3 cyp-3 Y75B12B.5	0.30	0.32	0.32	0.32	0.42	0.43	0.42	0.42	1.33
P02567	myo-1 R06C7.10	0.33	0.33	0.34	0.33	0.45	0.44	0.44	0.44	1.33
Q9U1Z4	CELE_Y60A3A.9 Y60A3A.9	0.41	0.00	0.23	0.22	0.38	0.00	0.48	0.29	1.33
O45011	CELE_W10C8.5 W10C8.5	0.31	0.33	0.31	0.32	0.41	0.42	0.45	0.42	1.33
Q09517	let-767 dhs-10 C56G2.6	0.36	0.32	0.31	0.33	0.44	0.45	0.43	0.44	1.33
Q9N4J2	vit-3 F59D8.1	0.32	0.28	0.30	0.30	0.40	0.41	0.39	0.40	1.33
Q9GYI1	CELE_F29B9.11 F29B9.11	0.52	0.26	0.27	0.35	0.47	0.49	0.45	0.47	1.34
Q21531	ifg-1 CELE_M110.4 M110.4	0.37	0.34	0.31	0.34	0.43	0.51	0.42	0.45	1.34
Q9GZH4	ribo-1 T22D1.4	0.36	0.31	0.30	0.32	0.43	0.42	0.45	0.43	1.34
O01816	cytb-5.2 CELE_W02D3.1 W02D3.1	0.30	0.28	0.31	0.30	0.36	0.42	0.42	0.40	1.34
P24894	ctc-2 coII cox-2 MTCE.31	0.29	0.27	0.34	0.30	0.40	0.41	0.39	0.40	1.34
Q18090	tomm-40 C18E9.6	0.30	0.31	0.34	0.32	0.44	0.41	0.43	0.43	1.34
Q2V4S2	gdi-1 CELE_Y57G11C.10 Y57G11C.10	0.32	0.44	0.32	0.36	0.46	0.53	0.46	0.48	1.35
Q86NH9	ttr-41 CELE_F10G7.11 F10G7.11	0.32	0.31	0.29	0.30	0.41	0.40	0.42	0.41	1.35
O61907	lin-40 CELE_T27C4.4 T27C4.4	0.32	0.00	0.00	0.11	0.44	0.00	0.00	0.15	1.35
Q9U307	gln-3 CELE_Y105C5B.28 Y105C5B.28	0.37	0.30	0.31	0.33	0.45	0.43	0.44	0.44	1.35
O02642	sucl-2 CELE_F23H11.3 F23H11.3	0.33	0.31	0.30	0.31	0.40	0.44	0.42	0.42	1.35
P54889	alh-13 T22H6.2	0.00	0.00	0.32	0.11	0.00	0.00	0.44	0.15	1.35
Q09450	C05C10.3	0.29	0.31	0.33	0.31	0.42	0.45	0.40	0.42	1.35
P54811	cdc-48.1 C06A1.1	0.00	0.32	0.30	0.21	0.00	0.42	0.42	0.28	1.35
Q23237	pbs-3 Y38A8.2	0.34	0.36	0.34	0.35	0.42	0.52	0.47	0.47	1.35
Q19286	ifb-2 F10C1.7	0.32	0.31	0.33	0.32	0.44	0.44	0.43	0.44	1.36
P50093	phb-2 T24H7.1	0.30	0.30	0.32	0.31	0.42	0.42	0.39	0.41	1.36
Q21770	wago-1 R06C7.1	0.31	0.00	0.00	0.10	0.42	0.00	0.00	0.14	1.36
Q20655	ftt-2 F52D10.3	0.32	0.33	0.31	0.32	0.44	0.44	0.42	0.43	1.36
O61199	ogdh-1 T22B11.5	0.32	0.31	0.33	0.32	0.43	0.44	0.43	0.43	1.36
Q20135	col-81 CELE_F38A3.1 F38A3.1	0.00	0.23	0.24	0.15	0.00	0.22	0.41	0.21	1.36
P48053	C05D11.1	0.00	0.32	0.00	0.11	0.00	0.44	0.00	0.15	1.36
Q95008	pas-5 F25H2.9	0.34	0.29	0.32	0.31	0.42	0.43	0.43	0.43	1.36
Q965Q1	CELE_Y22D7AL.10 Y22D7AL.10	0.32	0.30	0.33	0.32	0.42	0.43	0.44	0.43	1.36
Q02335	ZK370.8	0.34	0.00	0.00	0.11	0.46	0.00	0.00	0.15	1.36

Q18823	lam-2 C54D1.5	0.33	0.00	0.39	0.24	0.48	0.00	0.49	0.32	1.36
O44549	acdh-3 CELE_K06A5.6 K06A5.6	0.33	0.31	0.29	0.31	0.39	0.42	0.45	0.42	1.36
O44400	F37C4.5	0.33	0.31	0.32	0.32	0.41	0.44	0.45	0.44	1.36
O18239	sec-61 CELE_Y57G11C.15 Y57G11C.15	0.30	0.34	0.32	0.32	0.38	0.49	0.44	0.44	1.36
G5EDP2	daf-22 Y57A10C.6	0.30	0.47	0.29	0.35	0.44	0.50	0.50	0.48	1.36
P12845	myo-2 T18D3.4	0.32	0.32	0.32	0.32	0.44	0.44	0.44	0.44	1.36
O45713	CELE_R09B3.3 R09B3.3	0.32	0.29	0.32	0.31	0.51	0.39	0.38	0.43	1.37
Q19328	tsn-1 CELE_F10G7.2 F10G7.2	0.30	0.33	0.33	0.32	0.45	0.44	0.43	0.44	1.37
O45734	cpl-1 CELE_T03E6.7 T03E6.7	0.32	0.32	0.33	0.32	0.44	0.41	0.46	0.44	1.37
Q23158	atn-1 CELE_W04D2.1 W04D2.1	0.32	0.32	0.28	0.31	0.39	0.40	0.47	0.42	1.37
Q17832	vgln-1 C08H9.2 CELE_C08H9.2	0.30	0.32	0.33	0.32	0.44	0.44	0.43	0.44	1.37
Q9TYW1	vha-19 CELE_Y55H10A.1 Y55H10A.1	0.31	0.32	0.30	0.31	0.44	0.40	0.43	0.43	1.37
Q95XS2	CELE_Y38F2AR.9 Y38F2AR.9	0.43	0.00	0.42	0.28	0.57	0.00	0.58	0.39	1.37
Q94230	plp-1 CELE_F45E4.2 F45E4.2	0.31	0.33	0.31	0.32	0.44	0.43	0.44	0.43	1.37
G5EDD1	ucr-2.1 CELE_VW06B3R.1 VW06B3R.1	0.30	0.38	0.30	0.33	0.44	0.46	0.45	0.45	1.37
P91427	pgk-1 T03F1.3	0.31	0.32	0.31	0.31	0.43	0.41	0.44	0.43	1.37
G5EGK8	let-92 F38H4.9	0.00	0.00	0.30	0.10	0.00	0.00	0.41	0.14	1.37
Q21351	gtbp-1 CELE_K08F4.2 K08F4.2	0.31	0.30	0.30	0.30	0.42	0.40	0.42	0.42	1.37
Q19202	apy-1 F08C6.6	0.00	0.00	0.31	0.10	0.00	0.00	0.43	0.14	1.38
O17921	tbb-1 CELE_K01G5.7 K01G5.7	0.33	0.32	0.30	0.32	0.42	0.44	0.45	0.43	1.38
O44738	CELE_F57B10.5 F57B10.5	0.00	0.27	0.41	0.23	0.00	0.34	0.59	0.31	1.38
Q20779	tag-174 F54D8.2	0.30	0.32	0.32	0.31	0.45	0.42	0.42	0.43	1.38
Q17574	tag-165 C01G6.6	0.32	0.31	0.32	0.32	0.44	0.44	0.43	0.43	1.38
G5ECA7	CELE_T02D1.8 T02D1.8	0.00	0.00	0.32	0.11	0.00	0.00	0.44	0.15	1.38
Q20970	mrs-1 F58B3.5	0.30	0.30	0.34	0.31	0.44	0.43	0.42	0.43	1.38
Q19126	asb-2 CELE_F02E8.1 F02E8.1	0.31	0.30	0.32	0.31	0.43	0.44	0.44	0.43	1.38
B7WNA0	pyk-1 CELE_F25H5.3 F25H5.3	0.35	0.33	0.31	0.33	0.43	0.46	0.47	0.45	1.38
Q20684	lec-2 CELE_F52H3.7 F52H3.7	0.32	0.31	0.32	0.32	0.44	0.46	0.41	0.44	1.38
Q23655	nlt-1 ZK892.2	0.30	0.00	0.28	0.20	0.36	0.00	0.45	0.27	1.38
P55954	cco-2 Y37D8A.14	0.34	0.32	0.32	0.32	0.45	0.46	0.43	0.45	1.39
Q9TYK1	Y66H1A.4	0.31	0.34	0.34	0.33	0.46	0.51	0.41	0.46	1.39
O62102	pbs-2 C47B2.4 CELE_C47B2.4	0.36	0.42	0.00	0.26	0.47	0.61	0.00	0.36	1.39
G5EGK1	tln-1 CELE_Y71G12B.11 Y71G12B.11	0.28	0.00	0.00	0.09	0.39	0.00	0.00	0.13	1.39
Q9UAQ6	rab-1 C39F7.4 CELE_C39F7.4	0.33	0.30	0.30	0.31	0.41	0.43	0.45	0.43	1.39
Q9BKU4	phb-1 Y37E3.9	0.33	0.29	0.30	0.30	0.42	0.43	0.42	0.42	1.39
Q9U2D6	cnp-2 Y46G5A.10	0.00	0.00	0.30	0.10	0.00	0.00	0.42	0.14	1.39
Q22494	vha-15 T14F9.1	0.32	0.34	0.30	0.32	0.46	0.45	0.44	0.45	1.39
Q9NEN6	rps-6 Y71A12B.1	0.31	0.32	0.34	0.32	0.46	0.45	0.44	0.45	1.39
Q95Q60	fkb-5 C50F2.6 CELE_C50F2.6	0.47	0.25	0.36	0.36	0.46	0.49	0.54	0.50	1.39
Q86S57	alh-4 CELE_T05H4.13 T05H4.13	0.29	0.00	0.32	0.21	0.47	0.00	0.39	0.29	1.39
Q9XX57	dct-16 CELE_Y38H6C.1 Y38H6C.1	0.31	0.31	0.31	0.31	0.43	0.43	0.44	0.43	1.39
Q8WQA8	rps-20 CELE_Y105E8A.16 Y105E8A.16	0.31	0.31	0.32	0.31	0.44	0.44	0.44	0.44	1.39
O16249	CELE_F44E7.4 F44E7.4	0.32	0.31	0.35	0.33	0.45	0.45	0.47	0.46	1.39
Q18212	hel-1 C26D10.2	0.29	0.32	0.35	0.32	0.46	0.45	0.43	0.45	1.39
Q94246	gfi-1 CELE_F57F4.3 F57F4.3	0.30	0.30	0.33	0.31	0.44	0.43	0.42	0.43	1.40
G5EBJ7	fbp-1 fbp CELE_K07A3.1 K07A3.1	0.34	0.30	0.30	0.31	0.42	0.45	0.44	0.44	1.40
Q9N3B0	Y54G2A.23	0.29	0.32	0.31	0.31	0.40	0.43	0.45	0.43	1.40
Q20676	pccb-1 CELE_F52E4.1 F52E4.1	0.33	0.32	0.31	0.32	0.45	0.44	0.44	0.44	1.40
Q9XTI0	B0250.5	0.29	0.29	0.30	0.29	0.41	0.41	0.42	0.41	1.40
Q95X44	vha-8 C17H12.14 CELE_C17H12.14	0.31	0.32	0.32	0.32	0.44	0.44	0.44	0.44	1.40

Q18577	C42D4.1 CELE_C42D4.1	0.33	0.00	0.31	0.21	0.43	0.00	0.46	0.30	1.40
O76367	CELE_F29C4.2 F29C4.2	0.29	0.26	0.00	0.18	0.38	0.38	0.00	0.25	1.40
G5EE96	slc-25a10 slc-25A10 CELE_K11G12.5 K11G12.5	0.00	0.00	0.32	0.11	0.00	0.00	0.45	0.15	1.40
Q4W5P0	sqd-1 CELE_Y73B6BL.6 Y73B6BL.6	0.33	0.31	0.33	0.33	0.42	0.54	0.42	0.46	1.40
Q21746	sgt-1 CELE_R05F9.10 R05F9.10	0.32	0.31	0.32	0.32	0.43	0.46	0.45	0.45	1.40
Q22633	hpd-1 T21C12.2	0.30	0.37	0.31	0.32	0.47	0.43	0.46	0.45	1.40
Q9N5U1	CELE_T22F3.3 T22F3.3	0.31	0.33	0.30	0.31	0.44	0.45	0.42	0.44	1.40
Q8MNV8	C14B9.10 CELE_C14B9.10	0.00	0.27	0.32	0.20	0.00	0.44	0.40	0.28	1.40
Q7Z1Q3	alh-12 CELE_Y69F12A.2 Y69F12A.2	0.24	0.32	0.00	0.19	0.37	0.42	0.00	0.26	1.40
H2KZF5	pmt-1 CELE_ZK622.3 ZK622.3	0.33	0.35	0.30	0.33	0.44	0.46	0.47	0.46	1.40
Q10129	mrps-16 F56D1.3	0.00	0.30	0.00	0.10	0.00	0.42	0.00	0.14	1.40
Q95XI9	CELE_Y69A2AR.18 Y69A2AR.18	0.32	0.31	0.31	0.31	0.43	0.44	0.44	0.44	1.40
O01802	rpl-7 F53G12.10	0.31	0.32	0.34	0.32	0.45	0.45	0.44	0.45	1.40
Q20224	lbp-2 F40F4.2	0.32	0.36	0.28	0.32	0.43	0.45	0.47	0.45	1.40
B3WFT8	C14F11.4 CELE_C14F11.4	0.00	0.00	0.30	0.10	0.00	0.00	0.42	0.14	1.41
P11141	hsp-6 hsp70f C37H5.8	0.30	0.32	0.30	0.31	0.43	0.44	0.42	0.43	1.41
O62213	cey-1 CELE_F33A8.3 F33A8.3	0.33	0.33	0.33	0.33	0.45	0.46	0.47	0.46	1.41
Q9XTT9	rpt-6 CELE_Y49E10.1 Y49E10.1	0.42	0.35	0.30	0.35	0.49	0.49	0.52	0.50	1.41
G5EEK8	sca-1 mca-4 CELE_K11D9.2 K11D9.2	0.31	0.31	0.31	0.31	0.44	0.44	0.43	0.44	1.41
Q8MXS8	CELE_Y47G6A.22 Y47G6A.22	0.00	0.32	0.33	0.22	0.00	0.47	0.44	0.30	1.41
O18650	rps-19 T05F1.3	0.30	0.33	0.31	0.31	0.43	0.46	0.44	0.44	1.41
Q9XXK1	H28O16.1	0.30	0.32	0.31	0.31	0.44	0.43	0.45	0.44	1.41
P34328	hsp-12.2 hsp12-2 C14B9.1	0.33	0.29	0.31	0.31	0.45	0.43	0.43	0.44	1.41
G5EBP5	CELE_ZC247.1 ZC247.1	0.30	0.31	0.32	0.31	0.44	0.43	0.43	0.43	1.41
O01692	rps-17 T08B2.10	0.31	0.32	0.31	0.31	0.44	0.44	0.45	0.44	1.41
Q7K707	gpi-1 CELE_Y87G2A.8 Y87G2A.8	0.29	0.42	0.34	0.35	0.52	0.51	0.46	0.50	1.41
P54412	eef-1G F17C11.9	0.32	0.32	0.31	0.32	0.45	0.45	0.45	0.45	1.41
G5EFG4	abcf-2 CELE_T27E9.7 T27E9.7	0.30	0.32	0.32	0.32	0.42	0.48	0.44	0.45	1.41
P90762	rmd-2 C27H6.4 CELE_C27H6.4	0.31	0.31	0.30	0.31	0.43	0.43	0.44	0.43	1.42
P10299	gst-1 R107.7	0.33	0.31	0.37	0.34	0.48	0.51	0.44	0.48	1.42
H2KZ19	ampd-1 C34F11.3 CELE_C34F11.3	0.32	0.00	0.00	0.11	0.46	0.00	0.00	0.15	1.42
P90735	eat-6 B0365.3 CELE_B0365.3	0.31	0.32	0.30	0.31	0.44	0.44	0.44	0.44	1.42
O16517	atp-4 CELE_T05H4.12 T05H4.12	0.30	0.30	0.31	0.30	0.43	0.43	0.43	0.43	1.42
Q17763	atp-5 C06H2.1 CELE_C06H2.1	0.31	0.30	0.30	0.30	0.42	0.44	0.43	0.43	1.42
Q10576	dpy-18 phy-1 Y47D3B.10	0.41	0.28	0.29	0.33	0.38	0.53	0.49	0.47	1.42
Q09975	lys-8 C17G10.5 CELE_C17G10.5	0.30	0.00	0.00	0.10	0.43	0.00	0.00	0.14	1.42
P09588	his-3 T10C6.12; his-7 F45F2.4; his-12 ZK131.6; his-16 ZK131.10; his-19 K06C4.11; his-21 K06C4.3; his-30 F35H10.1; his-33 F17E9.13; his-43 F08G2.2; his-47 B0035.7; his-51 F07B7.10; his-53 F07B7.3; his-57 F54E12.5; his-61 F55G1.10; his-65 H02I12.7; his-68 T23D8.6	0.33	0.32	0.35	0.34	0.56	0.44	0.43	0.48	1.42
P28548	kin-10 kin-5 T01G9.6	0.33	0.00	0.00	0.11	0.47	0.00	0.00	0.16	1.42
Q18678	srs-2 C47E12.1	0.30	0.30	0.32	0.31	0.44	0.42	0.45	0.43	1.42
Q9NES7	CELE_Y39B6A.1 Y39B6A.1	0.24	0.27	0.30	0.27	0.36	0.41	0.39	0.39	1.42
Q21481	dhs-28 CELE_M03A8.1 M03A8.1	0.32	0.30	0.31	0.31	0.44	0.45	0.44	0.44	1.42
O45552	acaa-2 CELE_F53A2.7 F53A2.7	0.32	0.29	0.32	0.31	0.44	0.45	0.43	0.44	1.42
P53014	mlc-3 F09F7.2	0.31	0.31	0.31	0.31	0.44	0.44	0.44	0.44	1.42
P50880	rpl-3 F13B10.2	0.31	0.30	0.32	0.31	0.45	0.44	0.43	0.44	1.43
P34466	clu-1 F55H2.6	0.48	0.30	0.27	0.35	0.52	0.47	0.51	0.50	1.43
Q20585	rpn-7 F49C12.8	0.34	0.28	0.00	0.21	0.41	0.47	0.00	0.29	1.43
O16305	cmd-1 T21H3.3	0.30	0.33	0.31	0.31	0.44	0.46	0.44	0.44	1.43
P34460	eef-1B.1 F54H12.6	0.34	0.33	0.27	0.31	0.43	0.44	0.47	0.45	1.43

P49196	rps-12 F54E7.2	0.28	0.31	0.30	0.30	0.43	0.42	0.43	0.43	1.43
G5EDV3	cey-4 CELE_Y39A1C.3 Y39A1C.3	0.31	0.30	0.33	0.31	0.46	0.44	0.44	0.45	1.43
P06125	vit-5 C04F6.1	0.30	0.29	0.28	0.29	0.41	0.41	0.42	0.41	1.43
Q21525	CELE_M05D6.6 M05D6.6	0.30	0.00	0.30	0.20	0.45	0.00	0.41	0.29	1.43
Q23451	rad-23 CELE_ZK20.3 ZK20.3	0.00	0.33	0.00	0.11	0.00	0.47	0.00	0.16	1.43
Q09533	rpl-10 F10B5.1	0.31	0.33	0.31	0.32	0.45	0.46	0.45	0.45	1.43
Q21408	spc-1 CELE_K10B3.10 K10B3.10	0.36	0.27	0.32	0.32	0.48	0.44	0.46	0.46	1.43
P27639	inf-1 F57B9.6	0.34	0.29	0.30	0.31	0.43	0.45	0.45	0.44	1.43
Q21443	lmn-1 lam-1 DY3.2	0.32	0.33	0.33	0.33	0.47	0.46	0.47	0.47	1.43
Q9BL15	CELE_Y48G8AL.5 Y48G8AL.5	0.37	0.25	0.00	0.21	0.41	0.48	0.00	0.30	1.43
Q21750	aagr-2 CELE_R05F9.12 R05F9.12	0.28	0.00	0.00	0.09	0.41	0.00	0.00	0.14	1.43
P50432	mel-32 gly-1 C05D11.11	0.31	0.30	0.32	0.31	0.44	0.45	0.45	0.45	1.43
P17139	emb-9 clb-2 K04H4.1	0.31	0.28	0.32	0.30	0.44	0.44	0.42	0.43	1.43
Q22641	lpd-9 CELE_T21C9.5 T21C9.5	0.26	0.35	0.00	0.20	0.48	0.40	0.00	0.29	1.43
Q03577	drs-1 B0464.1	0.32	0.28	0.33	0.31	0.44	0.46	0.42	0.44	1.43
Q22716	rpl-32 CELE_T24B8.1 T24B8.1	0.30	0.31	0.31	0.31	0.44	0.43	0.44	0.44	1.43
Q9XWP7	eif-3.j eif-3.J CELE_Y40B1B.5 Y40B1B.5	0.33	0.25	0.35	0.31	0.47	0.42	0.45	0.45	1.44
G5ECM6	tufm-1 CELE_Y71H2AM.23 Y71H2AM.23	0.32	0.32	0.31	0.31	0.47	0.43	0.46	0.45	1.44
A7LPG5	cisd-1 CELE_W02B12.15 W02B12.15	0.28	0.34	0.29	0.30	0.44	0.43	0.43	0.43	1.44
Q19706	eif-3.G F22B5.2	0.36	0.00	0.53	0.30	0.39	0.60	0.29	0.43	1.44
Q19749	dlat-1 F23B12.5	0.29	0.31	0.31	0.30	0.42	0.43	0.45	0.43	1.44
O45815	act-5 CELE_T25C8.2 T25C8.2	0.30	0.30	0.32	0.31	0.44	0.45	0.44	0.44	1.44
Q9U2H9	eef-1B.2 Y41E3.10	0.31	0.31	0.31	0.31	0.43	0.45	0.46	0.45	1.44
Q95YF3	cgh-1 C07H6.5	0.30	0.29	0.32	0.30	0.42	0.44	0.45	0.44	1.44
Q09622	puf-12 ZK945.3	0.00	0.41	0.00	0.14	0.00	0.59	0.00	0.20	1.44
O01829	ssp-19 C55C2.2	0.32	0.30	0.23	0.28	0.37	0.44	0.42	0.41	1.44
Q9NA78	CELE_Y57A10A.23 Y57A10A.23	0.35	0.32	0.30	0.32	0.47	0.49	0.43	0.46	1.44
G5EEH6	ivd-1 C02B10.1 CELE_C02B10.1	0.31	0.26	0.29	0.29	0.42	0.40	0.43	0.42	1.44
P53013	eft-3 F31E3.5; eft-4 R03G5.1	0.31	0.31	0.30	0.31	0.44	0.44	0.44	0.44	1.44
Q9N4J8	cct-3 F54A3.3	0.32	0.31	0.31	0.31	0.46	0.44	0.45	0.45	1.44
O61820	eif-3.E B0511.10	0.00	0.34	0.32	0.22	0.00	0.46	0.48	0.31	1.44
Q9U348	col-93 CELE_W05B2.5 W05B2.5	0.30	0.31	0.34	0.32	0.46	0.45	0.46	0.46	1.44
Q93379	gsr-1 C46F11.2	0.36	0.29	0.29	0.32	0.49	0.45	0.43	0.45	1.44
Q21832	rnp-4 RBM8 Y14 CELE_R07E5.14 R07E5.14	0.00	0.27	0.00	0.09	0.00	0.39	0.00	0.13	1.44
Q22288	ttr-15 T07C4.5	0.30	0.28	0.31	0.30	0.44	0.42	0.43	0.43	1.44
Q27527	enol-1 T21B10.2	0.31	0.31	0.31	0.31	0.45	0.45	0.44	0.44	1.44
O45060	C35B1.5 CELE_C35B1.5	0.29	0.33	0.37	0.33	0.47	0.50	0.46	0.48	1.44
P36573	lec-1 W09H1.6	0.31	0.30	0.29	0.30	0.43	0.43	0.44	0.43	1.45
O44898	CELE_ZK484.5 ZK484.5	0.30	0.28	0.29	0.29	0.42	0.41	0.43	0.42	1.45
O61848	CELE_K03E5.2 K03E5.2	0.00	0.26	0.30	0.19	0.00	0.40	0.41	0.27	1.45
Q17361	usp-14 tgt-1 C13B4.2	0.00	0.36	0.26	0.21	0.00	0.43	0.48	0.30	1.45
P48150	rps-14 F37C12.9	0.30	0.33	0.32	0.31	0.46	0.46	0.45	0.45	1.45
Q27888	ldh-1 F13D12.2	0.27	0.33	0.32	0.31	0.41	0.37	0.55	0.44	1.45
Q23621	gdh-1 CELE_ZK829.4 ZK829.4	0.30	0.29	0.30	0.30	0.43	0.44	0.43	0.43	1.45
H9G2T4	idh-1 CELE_F59B8.2 F59B8.2	0.31	0.31	0.31	0.31	0.46	0.45	0.44	0.45	1.45
Q10454	F46H5.3	0.32	0.29	0.31	0.30	0.44	0.44	0.44	0.44	1.45
Q9N5B3	CELE_W08E12.7 W08E12.7	0.30	0.30	0.31	0.30	0.44	0.44	0.44	0.44	1.45
P34519	K11H3.3	0.28	0.30	0.35	0.31	0.47	0.44	0.44	0.45	1.45
Q7JNG1	atp-3 CELE_F27C1.7 F27C1.7	0.30	0.29	0.31	0.30	0.44	0.43	0.43	0.43	1.45
P34455	aco-2 F54H12.1	0.30	0.29	0.31	0.30	0.44	0.43	0.44	0.44	1.46

P49180	rpl-33 F10E7.7	0.32	0.33	0.30	0.31	0.46	0.46	0.46	0.46	1.46
P98080	ucr-1 F56D2.1	0.30	0.30	0.31	0.30	0.45	0.44	0.43	0.44	1.46
O61708	pqn-59 CELE_R119.4 R119.4	0.29	0.29	0.30	0.29	0.40	0.43	0.45	0.43	1.46
Q21824	prdx-3 R07E5.2	0.33	0.28	0.32	0.31	0.44	0.45	0.45	0.45	1.46
Q9N5U5	cdc-73 F35F11.1	0.30	0.00	0.00	0.10	0.44	0.00	0.00	0.15	1.46
P02566	unc-54 myo-4 F11C3.3	0.31	0.30	0.31	0.31	0.44	0.45	0.44	0.45	1.46
Q18853	cyc-1 C54G4.8 CELE_C54G4.8	0.30	0.29	0.32	0.30	0.44	0.44	0.45	0.44	1.46
Q9XXR4	ttr-24 CELE_Y51A2D.9 Y51A2D.9	0.24	0.00	0.25	0.16	0.39	0.00	0.33	0.24	1.46
G5EGP4	vha-6 CELE_VW02B12L.1 VW02B12L.1	0.00	0.00	0.41	0.14	0.00	0.00	0.59	0.20	1.46
Q18885	icd-1 C56C10.8	0.31	0.29	0.31	0.30	0.44	0.46	0.44	0.44	1.46
O18240	rps-18 CELE_Y57G11C.16 Y57G11C.16	0.31	0.30	0.31	0.31	0.44	0.44	0.46	0.45	1.46
Q18680	pyp-1 uba-1 C47E12.4	0.29	0.31	0.29	0.29	0.43	0.43	0.44	0.43	1.46
P37165	ubl-1 H06I04.4	0.31	0.31	0.30	0.31	0.45	0.45	0.45	0.45	1.46
Q20772	F54D5.7	0.00	0.29	0.29	0.19	0.00	0.41	0.43	0.28	1.46
P49181	rpl-36 F37C12.4	0.30	0.32	0.31	0.31	0.46	0.45	0.47	0.46	1.46
O17861	F37H8.5	0.28	0.28	0.30	0.28	0.44	0.40	0.42	0.42	1.46
P49029	mag-1 R09B3.5	0.00	0.31	0.27	0.19	0.00	0.41	0.44	0.28	1.47
Q9N4M4	anc-1 ZK973.6	0.31	0.31	0.31	0.31	0.46	0.45	0.45	0.45	1.47
Q22993	pmt-2 CELE_F54D11.1 F54D11.1	0.30	0.32	0.31	0.31	0.46	0.45	0.45	0.45	1.47
Q86D21	tars-1 C47D12.6 CELE_C47D12.6	0.31	0.29	0.28	0.30	0.42	0.43	0.46	0.44	1.47
Q17624	arrd-25 C04C11.2 CELE_C04C11.2	0.34	0.00	0.30	0.21	0.48	0.00	0.46	0.31	1.47
P0CG71	ubq-1 ubia F25B5.4	0.29	0.30	0.28	0.29	0.42	0.43	0.43	0.42	1.47
P91423	CELE_T03F1.11 T03F1.11	1.00	0.00	0.25	0.42	0.69	0.69	0.46	0.61	1.47
P52013	cyn-5 cyp-5 F31C3.1	0.31	0.29	0.30	0.30	0.45	0.43	0.43	0.44	1.47
Q22347	acdh-10 T08G2.3	0.41	0.29	0.28	0.33	0.56	0.44	0.44	0.48	1.47
P91134	adss-1 C37H5.6	0.34	0.32	0.31	0.32	0.45	0.55	0.43	0.48	1.47
Q19420	ttx-7 F13G3.5	0.35	0.26	0.33	0.31	0.42	0.47	0.49	0.46	1.47
Q19339	acs-14 CELE_F11A3.1 F11A3.1	0.00	0.28	0.31	0.20	0.00	0.42	0.45	0.29	1.47
Q20647	rpl-25.2 F52B5.6	0.29	0.31	0.31	0.30	0.45	0.45	0.44	0.45	1.47
P51404	rps-13 C16A3.9	0.31	0.31	0.31	0.31	0.46	0.45	0.45	0.46	1.47
H2L0I2	lfi-1 CELE_ZC8.4 ZC8.4	0.00	0.33	0.00	0.11	0.00	0.48	0.00	0.16	1.47
Q9BKU5	CELE_Y37E3.8 Y37E3.8	0.30	0.30	0.30	0.30	0.44	0.44	0.44	0.44	1.47
P91128	rpl-13 C32E8.2	0.32	0.29	0.31	0.31	0.45	0.46	0.45	0.45	1.47
P52015	cyn-7 cyp-7 Y75B12B.2	0.30	0.31	0.31	0.31	0.45	0.45	0.46	0.45	1.47
Q19087	dnpp-1 F01F1.9	0.29	0.32	0.35	0.32	0.47	0.50	0.45	0.48	1.47
P47207	cct-2 cctb T21B10.7	0.32	0.30	0.32	0.31	0.45	0.48	0.45	0.46	1.47
Q9TYY0	CELE_M57.2 M57.2	0.31	0.00	0.00	0.10	0.46	0.00	0.00	0.15	1.48
Q18943	CELE_D1054.10 D1054.10	0.39	0.28	0.31	0.32	0.49	0.51	0.43	0.48	1.48
Q966C6	rpl-7A Y24D9A.4	0.31	0.31	0.31	0.31	0.46	0.45	0.46	0.46	1.48
G5EGP8	cpz-1 CELE_F32B5.8 F32B5.8	0.32	0.29	0.32	0.31	0.44	0.46	0.47	0.46	1.48
D0IMZ5	fln-1 CELE_Y66H1B.2 Y66H1B.2	0.33	0.30	0.29	0.31	0.44	0.45	0.47	0.45	1.48
G5EGL2	CELE_T28D6.6 T28D6.6	0.00	0.00	0.29	0.10	0.00	0.00	0.43	0.14	1.48
O01541	aars-2 ars-2 F28H1.3	0.31	0.31	0.31	0.31	0.46	0.47	0.45	0.46	1.48
Q17633	C04G2.9 CELE_C04G2.9	0.29	0.00	0.26	0.18	0.40	0.00	0.42	0.27	1.48
Q07750	unc-60 C38C3.5	0.27	0.34	0.34	0.32	0.48	0.45	0.47	0.47	1.48
O62431	ers-1 qrs-5 Y41E3.4	0.32	0.26	0.33	0.30	0.43	0.48	0.44	0.45	1.48
Q19278	hach-1 CELE_F09F7.4 F09F7.4	0.31	0.32	0.31	0.31	0.46	0.47	0.47	0.47	1.48
P91871	fasn-1 CELE_F32H2.5 F32H2.5	0.32	0.36	0.30	0.32	0.48	0.47	0.49	0.48	1.48
O02108	cdc-37 W08F4.8	0.30	0.00	0.00	0.10	0.45	0.00	0.00	0.15	1.48
P34689	glh-1 T21G5.3	0.31	0.32	0.30	0.31	0.45	0.46	0.47	0.46	1.48

P46563	aldo-2 F01F1.12	0.31	0.30	0.30	0.30	0.45	0.45	0.45	0.45	1.48
P91253	gst-7 F11G11.2	0.29	0.31	0.35	0.32	0.53	0.44	0.43	0.47	1.48
O17406	attf-2 CELE_F09G2.9 F09G2.9	0.30	0.34	0.28	0.31	0.45	0.41	0.50	0.46	1.48
P61866	rpl-12 JC8.3	0.30	0.29	0.30	0.30	0.44	0.45	0.44	0.44	1.48
Q27389	rpl-16 M01F1.2	0.32	0.29	0.31	0.30	0.44	0.45	0.45	0.45	1.48
G5EBY3	nmy-2 CELE_F20G4.3 F20G4.3	0.00	0.00	0.25	0.08	0.00	0.00	0.38	0.13	1.48
Q9U3Q0	mrps-22 C14A4.14 CELE_C14A4.14	0.31	0.00	0.00	0.10	0.46	0.00	0.00	0.15	1.49
Q09544	F58F12.1	0.30	0.29	0.30	0.30	0.44	0.44	0.44	0.44	1.49
P52899	T05H10.6	0.28	0.31	0.31	0.30	0.45	0.44	0.46	0.45	1.49
G5EGA5	fat-2 W02A2.1	0.26	0.30	0.31	0.29	0.47	0.40	0.43	0.43	1.49
Q20938	rpn-6.1 F57B9.10	0.32	0.30	0.22	0.28	0.45	0.46	0.35	0.42	1.49
Q9U2M4	Y38F1A.6	0.28	0.30	0.33	0.30	0.48	0.45	0.43	0.45	1.49
Q22866	lev-11 tmy-1 Y105E8B.1	0.31	0.30	0.31	0.30	0.45	0.45	0.45	0.45	1.49
P10567	unc-15 F07A5.7	0.29	0.29	0.30	0.30	0.44	0.44	0.43	0.44	1.49
Q95005	pas-4 C36B1.4	0.30	0.30	0.30	0.30	0.45	0.44	0.43	0.44	1.49
Q9NAI5	CELE_Y39G8B.1 Y39G8B.1	0.31	0.31	0.29	0.30	0.43	0.46	0.45	0.45	1.49
Q19626	vha-12 F20B6.2	0.30	0.29	0.31	0.30	0.46	0.44	0.45	0.45	1.49
P52275	tbb-2 C36E8.5	0.31	0.30	0.31	0.31	0.45	0.46	0.46	0.46	1.49
P34575	cts-1 T20G5.2	0.29	0.29	0.29	0.29	0.44	0.41	0.45	0.43	1.49
Q19057	acdh-12 CELE_E04F6.5 E04F6.5	0.29	0.40	0.33	0.34	0.55	0.45	0.50	0.50	1.49
Q11176	unc-78 C04F6.4	0.32	0.30	0.30	0.31	0.44	0.45	0.48	0.46	1.49
Q18864	sft-4 surf-4 C54H2.5	0.00	0.40	0.00	0.13	0.00	0.60	0.00	0.20	1.49
P20163	hsp-4 hsp70d F43E2.8	0.29	0.29	0.30	0.29	0.44	0.43	0.46	0.44	1.49
Q09543	paa-1 F48E8.5	0.37	0.37	0.18	0.31	0.48	0.42	0.49	0.46	1.49
Q9U302	pab-1 CELE_Y106G6H.2 Y106G6H.2	0.30	0.30	0.32	0.31	0.46	0.46	0.45	0.46	1.49
Q17849	hsp-25 C09B8.6 CELE_C09B8.6	0.00	0.39	0.31	0.23	0.00	0.61	0.43	0.35	1.49
O45148	dlst-1 CELE_W02F12.5 W02F12.5	0.32	0.30	0.31	0.31	0.44	0.50	0.44	0.46	1.50
O01974	eif-3.H C41D11.2	0.35	0.24	0.40	0.33	0.42	0.54	0.52	0.49	1.50
H9G2P9	cbs-1 CELE_ZC373.1 ZC373.1	0.00	0.30	0.33	0.21	0.00	0.46	0.48	0.31	1.50
Q21732	CELE_R04F11.2 R04F11.2	0.29	0.29	0.30	0.29	0.44	0.44	0.43	0.44	1.50
O45319	tin-13 tim-13 DY3.1	0.31	0.26	0.31	0.29	0.42	0.46	0.44	0.44	1.50
Q23500	aco-1 gei-22 ZK455.1	0.31	0.30	0.31	0.31	0.47	0.45	0.46	0.46	1.50
Q19853	CELE_F28B4.3 F28B4.3	0.33	0.31	0.31	0.32	0.45	0.47	0.49	0.47	1.50
Q86S26	pmp-5 CELE_T10H9.5 T10H9.5	0.40	0.31	0.31	0.34	0.60	0.47	0.48	0.51	1.50
P41932	par-5 ftt-1 M117.2	0.29	0.31	0.31	0.30	0.45	0.45	0.46	0.46	1.50
Q20626	CELE_F49E2.5 F49E2.5	0.40	0.25	0.32	0.32	0.60	0.40	0.44	0.48	1.50
Q20053	asb-1 CELE_F35G12.10 F35G12.10	0.27	0.32	0.38	0.32	0.51	0.42	0.52	0.48	1.50
Q9BL03	CELE_Y54F10AM.5 Y54F10AM.5	0.30	0.28	0.26	0.28	0.43	0.37	0.45	0.42	1.50
G5EEK9	vha-5 CELE_F35H10.4 F35H10.4	0.32	0.29	0.00	0.20	0.45	0.47	0.00	0.31	1.50
P34334	rpl-21 C14B9.7	0.32	0.30	0.30	0.31	0.46	0.47	0.45	0.46	1.50
Q20203	csq-1 CELE_F40E10.3 F40E10.3	0.37	0.29	0.28	0.31	0.44	0.46	0.51	0.47	1.50
P49049	imp-2 T05E11.5	0.28	0.27	0.38	0.31	0.50	0.47	0.42	0.46	1.50
O45218	ads-1 Y50D7A.7	0.29	0.28	0.30	0.29	0.44	0.46	0.42	0.44	1.50
Q9U241	CELE_Y56A3A.19 Y56A3A.19	0.31	0.34	0.39	0.35	0.51	0.46	0.61	0.52	1.50
O01812	lbp-6 W02D3.5	0.32	0.29	0.31	0.31	0.46	0.47	0.45	0.46	1.50
Q19869	rpl-26 F28C6.7	0.31	0.30	0.31	0.31	0.45	0.45	0.48	0.46	1.50
Q22038	rho-1 rhoa Y51H4A.3	0.30	0.30	0.31	0.30	0.46	0.45	0.45	0.45	1.50
Q9N5E4	CELE_T02H6.11 T02H6.11	0.36	0.30	0.28	0.31	0.46	0.47	0.48	0.47	1.50
P91477	pbs-4 T20F5.2	0.35	0.00	0.27	0.21	0.50	0.00	0.43	0.31	1.50
O17759	tkt-1 CELE_F01G10.1 F01G10.1	0.28	0.32	0.31	0.30	0.45	0.47	0.45	0.46	1.50

Q19007	nap-1 CELE_D2096.8 D2096.8	0.30	0.30	0.30	0.30	0.45	0.45	0.45	0.45	1.50
P34559	ech-6 T05G5.6	0.29	0.29	0.30	0.29	0.44	0.45	0.44	0.44	1.50
G4SPY0	CELE_Y71G10AL.1 Y71G10AL.1	0.00	0.32	0.00	0.11	0.00	0.48	0.00	0.16	1.50
P50140	hsp-60 hsp60 Y22D7AL.5	0.30	0.29	0.30	0.30	0.44	0.46	0.45	0.45	1.51
O01504	rpa-2 C37A2.7	0.31	0.28	0.30	0.30	0.45	0.45	0.45	0.45	1.51
Q9TXI4	CELE_F23C8.5 F23C8.5	0.30	0.28	0.30	0.30	0.44	0.46	0.44	0.44	1.51
O01542	cpn-3 CELE_F28H1.2 F28H1.2	0.29	0.30	0.31	0.30	0.46	0.45	0.44	0.45	1.51
Q9XWS4	rpl-30 CELE_Y106G6H.3 Y106G6H.3	0.32	0.31	0.30	0.31	0.47	0.47	0.47	0.47	1.51
P54216	aldo-1 T05D4.1	0.31	0.28	0.30	0.30	0.44	0.45	0.46	0.45	1.51
P52819	rpl-22 C27A2.2	0.30	0.29	0.31	0.30	0.45	0.46	0.46	0.45	1.51
Q22021	R53.4	0.36	0.29	0.28	0.31	0.51	0.45	0.43	0.47	1.51
Q22620	pars-1 CELE_T20H4.3 T20H4.3	0.28	0.30	0.30	0.29	0.47	0.44	0.42	0.44	1.51
P51403	rps-2 C49H3.11	0.31	0.31	0.29	0.30	0.46	0.45	0.46	0.46	1.51
O02286	pck-2 CELE_R11A5.4 R11A5.4	0.30	0.29	0.29	0.29	0.45	0.45	0.43	0.44	1.51
Q21067	ifc-2 M6.1	0.36	0.30	0.30	0.32	0.51	0.45	0.48	0.48	1.51
Q93615	F27D4.1	0.29	0.29	0.30	0.29	0.44	0.45	0.43	0.44	1.51
H2KZG6	acdh-1 C55B7.4 CELE_C55B7.4	0.29	0.30	0.30	0.30	0.45	0.46	0.45	0.45	1.51
Q93573	tct-1 F25H2.11	0.30	0.30	0.29	0.30	0.45	0.45	0.44	0.45	1.51
Q9U2X0	prmt-1 CELE_Y113G7B.17 Y113G7B.17	0.29	0.29	0.30	0.29	0.44	0.45	0.43	0.44	1.51
P18948	vit-6 K07H8.6	0.28	0.27	0.28	0.28	0.42	0.42	0.42	0.42	1.51
O01806	C44E4.4 CELE_C44E4.4	0.30	0.24	0.33	0.29	0.45	0.44	0.43	0.44	1.51
P34382	far-1 F02A9.2	0.29	0.28	0.30	0.29	0.44	0.44	0.45	0.44	1.51
Q9N4H7	copa-1 CELE_Y71F9AL.17 Y71F9AL.17	0.30	0.31	0.27	0.29	0.47	0.45	0.41	0.44	1.51
Q20507	acbp-3 F47B10.7	0.29	0.29	0.31	0.30	0.48	0.44	0.43	0.45	1.51
P29691	eef-2 F25H5.4	0.30	0.30	0.30	0.30	0.45	0.46	0.44	0.45	1.52
Q9N3D9	nduf-5 CELE_Y54E10BL.5 Y54E10BL.5	0.41	0.23	0.24	0.29	0.36	0.47	0.51	0.45	1.52
Q93576	ndk-1 CELE_F25H2.5 F25H2.5	0.28	0.28	0.29	0.28	0.44	0.42	0.43	0.43	1.52
Q21233	nuo-4 CELE_K04G7.4 K04G7.4	0.32	0.28	0.31	0.30	0.56	0.37	0.44	0.46	1.52
O17643	idh-2 C34F6.8 CELE_C34F6.8	0.00	0.40	0.00	0.13	0.00	0.60	0.00	0.20	1.52
Q22100	kat-1 T02G5.8	0.31	0.26	0.37	0.31	0.54	0.48	0.42	0.48	1.52
G5EC91	dpy-11 CELE_F46E10.9 F46E10.9	0.31	0.30	0.27	0.29	0.43	0.45	0.45	0.44	1.52
P53588	suca-1 F47B10.1	0.32	0.31	0.28	0.31	0.46	0.50	0.42	0.46	1.52
O17214	fum-1 H14A12.2	0.30	0.32	0.28	0.30	0.46	0.44	0.47	0.46	1.52
Q9UAV5	mdh-1 CELE_F46E10.10 F46E10.10	0.30	0.30	0.29	0.30	0.44	0.46	0.46	0.45	1.52
Q21193	pfn-3 K03E6.6	0.00	0.00	0.30	0.10	0.00	0.00	0.45	0.15	1.52
O17536	hil-4 C18G1.5	0.30	0.30	0.34	0.31	0.49	0.48	0.45	0.47	1.52
G5ED07	pdi-3 CELE_H06O01.1 H06O01.1	0.30	0.30	0.30	0.30	0.45	0.46	0.46	0.46	1.52
Q9N3X2	rps-4 Y43B11AR.4	0.28	0.28	0.31	0.29	0.45	0.44	0.44	0.44	1.52
Q20719	F53F4.10	0.31	0.29	0.28	0.29	0.42	0.49	0.44	0.45	1.53
O62277	dct-18 CELE_F58G1.4 F58G1.4	0.29	0.29	0.28	0.29	0.45	0.43	0.43	0.44	1.53
Q27371	mup-2 T22E5.5	0.29	0.28	0.35	0.31	0.48	0.47	0.44	0.47	1.53
O17271	heh-1 R148.6	0.30	0.38	0.33	0.34	0.42	0.64	0.50	0.52	1.53
H2KYR1	vig-1 CELE_F56D12.5 F56D12.5	0.31	0.28	0.31	0.30	0.45	0.48	0.45	0.46	1.53
P27420	hsp-3 hsp70c C15H9.6	0.32	0.29	0.29	0.30	0.46	0.45	0.47	0.46	1.53
G1K0V8	iff-1 CELE_T05G5.10 T05G5.10	0.28	0.30	0.00	0.19	0.42	0.47	0.00	0.30	1.53
O62146	F09B12.3	0.00	0.30	0.25	0.18	0.00	0.50	0.35	0.28	1.53
G5EC31	alh-6 CELE_F56D12.1 F56D12.1	0.31	0.31	0.29	0.30	0.47	0.47	0.45	0.46	1.53
Q18421	C34C12.8	0.28	0.24	0.34	0.29	0.47	0.46	0.39	0.44	1.53
Q18688	daf-21 C47E8.5	0.30	0.30	0.30	0.30	0.46	0.47	0.46	0.46	1.53
Q21465	zig-12 CELE_M02D8.1 M02D8.1	0.00	0.28	0.33	0.20	0.00	0.46	0.46	0.31	1.53

O01868	rpl-24.1 D1007.12	0.28	0.29	0.28	0.28	0.43	0.44	0.44	0.44	1.54
H2KYQ5	gyg-1 F56B6.4	0.31	0.00	0.00	0.10	0.47	0.00	0.00	0.16	1.54
Q966C7	tald-1 CELE_Y24D9A.8 Y24D9A.8	0.31	0.29	0.29	0.29	0.45	0.45	0.46	0.45	1.54
Q9XW92	vha-13 Y49A3A.2	0.30	0.29	0.29	0.29	0.45	0.45	0.45	0.45	1.54
Q20780	alh-1 CELE_F54D8.3 F54D8.3	0.29	0.31	0.29	0.30	0.46	0.45	0.45	0.45	1.54
Q9GYK2	C05D9.3	0.39	0.00	0.00	0.13	0.61	0.00	0.00	0.20	1.54
H2KZS2	CELE_T23E7.2 T23E7.2	0.32	0.28	0.26	0.29	0.43	0.47	0.44	0.44	1.54
Q8MNU8	C29E4.12	0.27	0.32	0.00	0.20	0.48	0.43	0.00	0.31	1.54
O45444	clec-63 CELE_F35C5.6 F35C5.6	0.29	0.28	0.31	0.29	0.48	0.42	0.45	0.45	1.54
Q9GRZ9	tcc-1 CELE_Y59A8A.3 Y59A8A.3	0.21	0.00	0.26	0.16	0.38	0.00	0.34	0.24	1.54
O16462	grd-5 CELE_F41E6.2 F41E6.2	0.28	0.31	0.27	0.29	0.45	0.42	0.46	0.44	1.54
P27798	crt-1 Y38A10A.5	0.30	0.29	0.29	0.29	0.44	0.45	0.46	0.45	1.54
O17732	pyc-1 D2023.2	0.28	0.27	0.33	0.29	0.53	0.40	0.42	0.45	1.54
P91020	C07D8.6 CELE_C07D8.6	0.31	0.27	0.30	0.29	0.43	0.45	0.47	0.45	1.54
Q17967	pdi-1 C14B1.1	0.29	0.28	0.29	0.29	0.45	0.45	0.45	0.45	1.54
Q65ZK0	rars-1 CELE_F26F4.10 F26F4.10	0.27	0.31	0.34	0.30	0.53	0.46	0.43	0.47	1.54
Q19289	ifb-1 F10C1.2	0.30	0.30	0.30	0.30	0.46	0.47	0.46	0.46	1.55
P17329	gpd-2 K10B3.8	0.28	0.31	0.30	0.30	0.47	0.46	0.45	0.46	1.55
O17626	C31C9.2 CELE_C31C9.2	0.25	0.32	0.28	0.29	0.45	0.44	0.44	0.44	1.55
Q94269	CELE_K10C2.1 K10C2.1	0.32	0.40	0.30	0.34	0.46	0.60	0.51	0.53	1.55
Q86S66	icd-2 Y65B4BR.5	0.30	0.29	0.31	0.30	0.46	0.45	0.47	0.46	1.55
P52713	alh-8 F13D12.4	0.30	0.29	0.30	0.30	0.45	0.47	0.46	0.46	1.55
P27604	ahcy-1 ahh dpy-14 K02F2.2	0.29	0.29	0.29	0.29	0.46	0.46	0.45	0.45	1.55
Q23315	ears-1 CELE_ZC434.5 ZC434.5	0.29	0.36	0.27	0.31	0.53	0.42	0.48	0.48	1.55
Q95Y90	rpl-9 R13A5.8	0.29	0.30	0.28	0.29	0.45	0.46	0.45	0.45	1.55
O44989	col-49 CELE_K09H9.3 K09H9.3	0.20	0.11	0.15	0.15	0.22	0.24	0.25	0.24	1.55
Q20412	F44G4.2	0.00	0.27	0.31	0.19	0.00	0.44	0.45	0.30	1.55
O45293	gly-8 Y66A7A.6	0.00	0.00	0.30	0.10	0.00	0.00	0.47	0.16	1.55
G5EDZ9	cpi-1 CELE_K08B4.6 K08B4.6	0.39	0.00	0.00	0.13	0.61	0.00	0.00	0.20	1.55
Q18803	asg-2 C53B7.4	0.29	0.30	0.30	0.30	0.49	0.47	0.42	0.46	1.55
O17680	sams-1 C49F5.1	0.30	0.30	0.31	0.30	0.47	0.48	0.46	0.47	1.55
O45622	erfa-3 CELE_H19N07.1 H19N07.1	0.30	0.29	0.29	0.29	0.46	0.47	0.44	0.45	1.55
Q18787	rpt-1 C52E4.4	0.00	0.27	0.30	0.19	0.00	0.45	0.43	0.30	1.55
Q05036	hsp-110 C30C11.4	0.30	0.30	0.29	0.30	0.46	0.47	0.47	0.47	1.55
Q9NEW6	rsp-3 Y111B2A.18	0.24	0.31	0.22	0.26	0.41	0.42	0.37	0.40	1.56
Q17761	T25B9.9	0.33	0.27	0.30	0.30	0.45	0.48	0.45	0.46	1.56
P46561	atp-2 C34E10.6	0.30	0.27	0.28	0.29	0.44	0.45	0.44	0.45	1.56
O44156	pas-6 CD4.6	0.28	0.29	0.29	0.29	0.42	0.47	0.45	0.45	1.56
Q09261	mrps-31 C32A3.2	0.29	0.00	0.00	0.10	0.45	0.00	0.00	0.15	1.56
Q2L6Y6	hsp-75 CELE_R151.7 R151.7	0.29	0.00	0.00	0.10	0.45	0.00	0.00	0.15	1.56
Q21752	vdac-1 R05G6.7	0.30	0.30	0.29	0.29	0.46	0.45	0.46	0.46	1.56
G5EEE5	elo-1 ceelo1 CELE_F56H11.4 F56H11.4	0.29	0.30	0.30	0.29	0.45	0.48	0.45	0.46	1.56
O44480	rpl-20 E04A4.8	0.28	0.29	0.31	0.29	0.45	0.47	0.45	0.46	1.56
P04255	his-11 ZK131.5; his-15 ZK131.9; his-29 F35H10.11; his-34 F17E9.9; his-44 F08G2.1	0.29	0.28	0.30	0.29	0.45	0.46	0.45	0.45	1.56
Q21215	rack-1 K04D7.1	0.28	0.30	0.30	0.29	0.46	0.46	0.46	0.46	1.56
P41938	B0272.3	0.29	0.24	0.30	0.28	0.41	0.41	0.48	0.44	1.56
Q20206	rps-11 CELE_F40F11.1 F40F11.1	0.29	0.29	0.28	0.29	0.46	0.44	0.44	0.45	1.56
Q9GZE9	ldp-1 CELE_F22F7.1 F22F7.1	0.32	0.25	0.31	0.29	0.41	0.56	0.41	0.46	1.57
Q95YD5	vha-16 C30F8.2 CELE_C30F8.2	0.27	0.26	0.33	0.29	0.44	0.46	0.46	0.45	1.57
O16264	F40A3.3	0.32	0.30	0.31	0.31	0.44	0.57	0.44	0.48	1.57

Q9XUS5	CELE_K08E3.5 K08E3.5	0.23	0.00	0.00	0.08	0.36	0.00	0.00	0.12	1.57
Q20228	rps-9 F40F8.10	0.29	0.30	0.29	0.29	0.47	0.45	0.46	0.46	1.57
Q9N599	pas-3 Y110A7A.14	0.31	0.32	0.29	0.31	0.45	0.44	0.54	0.48	1.57
O76840	**mig-6 ppn-1 C37C3.6**	0.28	0.28	0.29	0.28	0.47	0.43	0.44	0.44	1.57
O17218	rps-22 CELE_F53A3.3 F53A3.3	0.32	0.30	0.29	0.30	0.48	0.46	0.47	0.47	1.57
P91374	rpl-15 K11H12.2	0.29	0.31	0.29	0.30	0.47	0.46	0.47	0.47	1.57
O44906	pck-1 CELE_W05G11.6 W05G11.6	0.28	0.25	0.30	0.28	0.40	0.45	0.46	0.43	1.57
O01761	unc-89 C09D1.1	0.30	0.28	0.29	0.29	0.44	0.46	0.45	0.45	1.57
O02158	CELE_T09B4.8 T09B4.8	0.00	0.30	0.00	0.10	0.00	0.46	0.00	0.15	1.57
H2KYJ5	mtch-1 CELE_F43E2.7 F43E2.7	0.24	0.37	0.33	0.31	0.51	0.52	0.44	0.49	1.57
P52717	F41C3.5	0.29	0.29	0.28	0.29	0.46	0.44	0.44	0.45	1.57
P91913	rla-1 rpa-1 Y37E3.7	0.30	0.29	0.28	0.29	0.46	0.45	0.47	0.46	1.57
P90868	pbs-7 CELE_F39H11.5 F39H11.5	0.25	0.00	0.32	0.19	0.45	0.00	0.44	0.30	1.57
O45865	ant-1.1 CELE_T27E9.1 T27E9.1	0.29	0.28	0.30	0.29	0.45	0.46	0.45	0.45	1.57
Q95QW0	eif-3.L C17G10.9	0.00	0.31	0.31	0.21	0.00	0.52	0.46	0.33	1.57
Q9U1X9	rla-2 CELE_Y62E10A.1 Y62E10A.1	0.27	0.27	0.29	0.28	0.42	0.43	0.45	0.43	1.57
O16309	fkb-3 C05C8.3 CELE_C05C8.3	0.29	0.24	0.28	0.27	0.44	0.38	0.46	0.43	1.57
Q9U1Q4	vrs-2 Y87G2A.5	0.29	0.31	0.29	0.30	0.46	0.47	0.47	0.47	1.57
P48156	rps-8 F42C5.8	0.28	0.28	0.30	0.29	0.45	0.46	0.45	0.45	1.58
Q9N4I4	rpl-10a rpl-1 Y71F9AL.13	0.28	0.29	0.28	0.28	0.44	0.45	0.45	0.45	1.58
Q27487	ctl-2 cat cat-1 Y54G11A.5	0.27	0.24	0.28	0.26	0.44	0.38	0.41	0.41	1.58
P34496	mtss-1 PAR2.1	0.30	0.00	0.00	0.10	0.48	0.00	0.00	0.16	1.58
P46769	rps-0 B0393.1	0.30	0.29	0.29	0.29	0.46	0.46	0.46	0.46	1.58
O45946	rpl-18 Y45F10D.12	0.29	0.28	0.29	0.29	0.45	0.45	0.45	0.45	1.58
O17586	pas-1 C15H11.7	0.32	0.29	0.26	0.29	0.46	0.44	0.47	0.46	1.58
O45499	rps-26 F39B2.6	0.29	0.28	0.29	0.29	0.45	0.46	0.46	0.45	1.58
P48162	rpl-25.1 F55D10.2	0.29	0.31	0.30	0.30	0.48	0.47	0.48	0.47	1.58
P91917	ola-1 tag-210 W08E3.3	0.28	0.27	0.30	0.28	0.46	0.44	0.44	0.45	1.58
O02266	alh-7 CELE_F45H10.1 F45H10.1	0.29	0.00	0.00	0.10	0.46	0.00	0.00	0.15	1.58
Q09581	lec-3 ZK892.1	0.00	0.34	0.27	0.20	0.00	0.47	0.49	0.32	1.58
P34383	far-2 F02A9.3	0.27	0.29	0.27	0.28	0.43	0.44	0.44	0.44	1.58
Q27493	rpoa-2 CELE_F14B4.3 F14B4.3	0.30	0.00	0.00	0.10	0.47	0.00	0.00	0.16	1.58
P34500	ttr-2 K03H1.4	0.29	0.30	0.31	0.30	0.50	0.46	0.47	0.47	1.58
G5EEA8	nex-1 NEX-1 CELE_ZC155.1 ZC155.1	0.29	0.29	0.29	0.29	0.45	0.47	0.45	0.46	1.58
O44750	app-1 W03G9.4	0.26	0.00	0.32	0.19	0.47	0.00	0.45	0.31	1.58
O02639	rpl-19 C09D4.5	0.32	0.28	0.29	0.29	0.43	0.45	0.52	0.47	1.58
O17528	sel-9 W02D7.7	0.30	0.27	0.28	0.28	0.45	0.44	0.45	0.45	1.58
Q9N5V3	imb-3 C53D5.6 CELE_C53D5.6	0.27	0.30	0.30	0.29	0.45	0.47	0.46	0.46	1.58
Q9BKP8	C17F4.7 CELE_C17F4.7	0.28	0.31	0.27	0.29	0.45	0.45	0.47	0.46	1.58
Q17993	C14F11.6 CELE_C14F11.6	0.00	0.31	0.00	0.10	0.00	0.49	0.00	0.16	1.58
P34662	rpl-35 ZK652.4	0.26	0.29	0.29	0.28	0.45	0.43	0.45	0.44	1.58
P41994	rpia-1 B0280.3	0.27	0.28	0.27	0.27	0.43	0.45	0.42	0.43	1.58
O76449	CELE_ZK1055.7 ZK1055.7	0.32	0.29	0.00	0.20	0.42	0.55	0.00	0.32	1.58
Q9GYF1	unc-27 tni-2 ZK721.2	0.30	0.28	0.31	0.29	0.46	0.48	0.47	0.47	1.58
Q21926	irs-1 R11A8.6	0.29	0.30	0.30	0.30	0.47	0.48	0.47	0.47	1.59
P48152	rps-3 C23G10.3	0.29	0.29	0.29	0.29	0.45	0.47	0.45	0.46	1.59
Q22370	ucr-2.2 CELE_T10B10.2 T10B10.2	0.26	0.29	0.28	0.28	0.44	0.44	0.44	0.44	1.59
Q19246	dhs-25 CELE_F09E10.3 F09E10.3	0.29	0.26	0.34	0.30	0.45	0.50	0.46	0.47	1.59
G5EEI4	asp-1 CELE_Y39B6A.20 Y39B6A.20	0.24	0.24	0.24	0.24	0.38	0.39	0.38	0.38	1.59
P34690	tba-2 C47B2.3	0.28	0.27	0.28	0.27	0.44	0.44	0.43	0.44	1.59

Q23588	upp-1 ZK783.2	0.25	0.00	0.00	0.08	0.40	0.00	0.00	0.13	1.59
Q7Z072	tnt-2 CELE_F53A9.10 F53A9.10	0.29	0.29	0.29	0.29	0.46	0.46	0.46	0.46	1.59
P37806	unc-87 F08B6.4	0.28	0.30	0.28	0.29	0.48	0.44	0.46	0.46	1.59
Q8WSN3	spp-17 C54G6.5 CELE_C54G6.5	0.00	0.00	0.31	0.10	0.00	0.00	0.49	0.16	1.59
O18000	pes-9 CELE_R11H6.1 R11H6.1	0.28	0.28	0.30	0.29	0.47	0.45	0.45	0.46	1.59
O45012	nol-5 CELE_W01B11.3 W01B11.3	0.30	0.28	0.30	0.29	0.48	0.47	0.46	0.47	1.59
Q93831	CELE_F59C6.5 F59C6.5	0.28	0.27	0.31	0.28	0.47	0.47	0.42	0.45	1.60
Q93619	tag-173 CELE_F27D4.5 F27D4.5	0.27	0.25	0.29	0.27	0.40	0.34	0.55	0.43	1.60
O17919	K01G5.5	0.27	0.30	0.30	0.29	0.46	0.47	0.46	0.46	1.60
Q93934	CELE_R07H5.8 R07H5.8	0.29	0.30	0.29	0.29	0.47	0.46	0.46	0.46	1.60
P53589	sucg-1 C50F7.4	0.30	0.28	0.30	0.29	0.48	0.45	0.47	0.47	1.60
Q9N5S7	CELE_F49H12.5 F49H12.5	0.29	0.26	0.30	0.28	0.46	0.48	0.42	0.45	1.60
P12844	myo-3 mhcA K12F2.1	0.30	0.31	0.29	0.30	0.49	0.48	0.48	0.49	1.60
Q93572	rpa-0 F25H2.10	0.29	0.29	0.29	0.29	0.47	0.47	0.46	0.46	1.60
Q17474	B0334.3 CELE_B0334.3	0.00	0.28	0.26	0.18	0.00	0.41	0.45	0.29	1.60
Q9TXU7	eif-1.a eif-1.A CELE_H06H21.3 H06H21.3	0.30	0.29	0.27	0.29	0.46	0.45	0.48	0.46	1.61
O44503	CELE_R02D3.1 R02D3.1	0.26	0.00	0.27	0.18	0.38	0.00	0.46	0.28	1.61
Q18310	C29F5.1 CELE_C29F5.1	0.00	0.32	0.28	0.20	0.00	0.49	0.47	0.32	1.61
Q21735	ran-4 R05D11.3	0.29	0.00	0.28	0.19	0.47	0.00	0.45	0.30	1.61
Q95Y28	CELE_Y34B4A.9 Y34B4A.9	0.00	0.00	0.28	0.09	0.00	0.00	0.45	0.15	1.61
Q22099	krs-1 T02G5.9	0.29	0.28	0.27	0.28	0.45	0.46	0.43	0.45	1.61
O01833	hil-5 B0414.3	0.33	0.31	0.19	0.28	0.41	0.42	0.50	0.44	1.61
Q22562	CELE_T19B10.2 T19B10.2	0.30	0.29	0.26	0.28	0.43	0.44	0.48	0.45	1.61
P05690	vit-2 C42D8.2	0.26	0.27	0.27	0.27	0.43	0.44	0.44	0.44	1.61
Q21219	pept-1 cptb opt-2 pep-2 K04E7.2	0.00	0.48	0.00	0.16	0.00	0.77	0.00	0.26	1.61
Q9N3H3	CELE_Y53G8AL.2 Y53G8AL.2	0.27	0.00	0.29	0.19	0.46	0.00	0.45	0.30	1.61
O44509	CELE_F42G8.10 F42G8.10	0.27	0.00	0.00	0.09	0.43	0.00	0.00	0.14	1.61
Q94261	cif-1 csn-7 eif-3.M K08F11.3	0.28	0.26	0.35	0.29	0.45	0.50	0.48	0.48	1.61
O45418	fkb-6 CELE_F31D4.3 F31D4.3	0.27	0.25	0.34	0.29	0.45	0.50	0.44	0.46	1.61
H2L0Q1	CELE_ZK6.11 ZK6.11	0.30	0.29	0.29	0.29	0.45	0.49	0.48	0.47	1.61
O01869	rps-10 CELE_D1007.6 D1007.6	0.30	0.29	0.29	0.29	0.48	0.47	0.46	0.47	1.62
G8JXY2	mthf-1 C06A8.1	0.31	0.31	0.30	0.30	0.47	0.50	0.51	0.49	1.62
P18947	vit-4 F59D8.2	0.28	0.26	0.27	0.27	0.44	0.44	0.43	0.44	1.62
P55326	F13E6.1	0.28	0.31	0.35	0.32	0.53	0.53	0.47	0.51	1.62
Q17880	nuo-1 C09H10.3 CELE_C09H10.3	0.27	0.26	0.29	0.27	0.45	0.44	0.44	0.44	1.62
Q19416	dylt-1 CELE_F13G3.4 F13G3.4	0.00	0.00	0.27	0.09	0.00	0.00	0.43	0.14	1.62
Q18785	mif-2 C52E4.2	0.29	0.55	0.00	0.28	0.40	0.43	0.53	0.45	1.62
Q93168	C01G10.8 CELE_C01G10.8	0.27	0.28	0.28	0.28	0.42	0.49	0.43	0.45	1.62
Q09486	C30G12.2 CELE_C30G12.2	0.00	0.28	0.00	0.09	0.00	0.45	0.00	0.15	1.62
G5EFZ1	ipgm-1 F57B10.3	0.30	0.29	0.30	0.30	0.46	0.47	0.52	0.48	1.62
Q9XVS4	dao-5 C25A1.10 CELE_C25A1.10	0.38	0.00	0.00	0.13	0.62	0.00	0.00	0.21	1.62
Q20720	CELE_F53F4.11 F53F4.11	0.30	0.00	0.27	0.19	0.47	0.00	0.45	0.31	1.62
G5EC10	lec-9 C16H3.2 CELE_C16H3.2	0.00	0.28	0.28	0.19	0.00	0.46	0.46	0.31	1.63
Q9GYT0	ech-1.2 CELE_T08B2.7 T08B2.7	0.30	0.29	0.26	0.28	0.45	0.45	0.48	0.46	1.63
P09446	hsp-1 hsp70a F26D10.3	0.28	0.27	0.28	0.28	0.46	0.45	0.45	0.45	1.63
Q18040	C16A3.10	0.29	0.28	0.29	0.28	0.47	0.47	0.46	0.46	1.63
O17694	ril-1 C53A5.1 CELE_C53A5.1	0.00	0.28	0.00	0.09	0.00	0.45	0.00	0.15	1.63
P47208	cct-4 K01C8.10	0.28	0.28	0.30	0.29	0.48	0.47	0.46	0.47	1.63
P49596	ppm-2 T23F11.1	0.27	0.00	0.00	0.09	0.45	0.00	0.00	0.15	1.63
O02640	mdh-2 F20H11.3	0.28	0.25	0.31	0.28	0.46	0.46	0.45	0.46	1.63

Q9N358	cct-8 Y55F3AR.3	0.30	0.28	0.31	0.30	0.47	0.49	0.50	0.49	1.63
G5EC23	hcf-1 C46A5.9 CELE_C46A5.9	0.14	0.00	0.43	0.19	0.58	0.00	0.35	0.31	1.63
Q10657	tpi-1 Y17G7B.7	0.29	0.29	0.29	0.29	0.48	0.46	0.46	0.47	1.63
Q9XVE9	rpl-14 C04F12.4 CELE_C04F12.4	0.28	0.27	0.28	0.27	0.45	0.45	0.45	0.45	1.63
Q9NF11	hprt-1 CELE_Y105E8B.5 Y105E8B.5	0.31	0.30	0.32	0.31	0.52	0.55	0.46	0.51	1.63
P49041	rps-5 T05E11.1	0.28	0.29	0.28	0.28	0.47	0.46	0.46	0.46	1.63
P54812	cdc-48.2 C41C4.8	0.29	0.30	0.28	0.29	0.50	0.47	0.45	0.48	1.64
O44451	pdhb-1 C04C3.3	0.27	0.30	0.30	0.29	0.47	0.51	0.45	0.48	1.64
Q86FL8	spp-5 CELE_T08A9.9 T08A9.9	0.28	0.25	0.00	0.18	0.43	0.43	0.00	0.29	1.64
Q19877	rps-23 F28D1.7	0.33	0.27	0.29	0.29	0.52	0.45	0.49	0.48	1.64
P41956	mev-1 cyt-1 T07C4.7	0.28	0.31	0.29	0.30	0.48	0.53	0.45	0.49	1.64
Q20122	CELE_F37C12.3 F37C12.3	0.26	0.00	0.00	0.09	0.43	0.00	0.00	0.14	1.64
Q09444	ubh-4 C08B11.7	0.31	0.00	0.22	0.18	0.46	0.00	0.41	0.29	1.64
G5EE04	hip-1 CELE_T12D8.8 T12D8.8	0.28	0.34	0.29	0.31	0.49	0.52	0.50	0.50	1.64
G5EDE8	ile-1 CELE_K07A1.8 K07A1.8	0.29	0.00	0.00	0.10	0.48	0.00	0.00	0.16	1.64
P47991	rpl-6 R151.3	0.28	0.28	0.29	0.28	0.47	0.46	0.46	0.46	1.64
Q21898	vha-1 R10E11.8	0.28	0.00	0.00	0.09	0.47	0.00	0.00	0.16	1.64
H2KZL7	prdx-2 CELE_F09E5.15 F09E5.15	0.26	0.29	0.29	0.28	0.44	0.48	0.45	0.46	1.65
Q17994	got-2.2 C14F11.1 CELE_C14F11.1	0.28	0.29	0.29	0.29	0.49	0.48	0.46	0.48	1.65
G5EFI4	lec-5 CELE_ZK1248.16 ZK1248.16	0.27	0.25	0.31	0.28	0.47	0.46	0.45	0.46	1.65
Q9GRY9	CELE_Y59A8B.10 Y59A8B.10	0.00	0.36	0.32	0.23	0.00	0.44	0.68	0.37	1.65
P49405	rpl-5 F54C9.5	0.29	0.28	0.29	0.29	0.46	0.47	0.49	0.47	1.65
Q4TT88	pam-1 CELE_F49E8.3 F49E8.3	0.28	0.29	0.30	0.29	0.51	0.45	0.48	0.48	1.65
G4SLH0	ttn-1 W06H8.8	0.28	0.30	0.26	0.28	0.46	0.45	0.47	0.46	1.65
Q20819	tsfm-1 F55C5.5	0.00	0.00	0.27	0.09	0.00	0.00	0.44	0.15	1.66
P40614	F01G4.6	0.28	0.28	0.28	0.28	0.47	0.47	0.46	0.46	1.66
Q22799	dlc-1 T26A5.9	0.28	0.29	0.28	0.28	0.47	0.47	0.46	0.47	1.66
Q95ZS5	CELE_F56A8.3 F56A8.3	0.28	0.30	0.27	0.28	0.45	0.49	0.46	0.47	1.66
A7LPE6	gpdh-2 CELE_K11H3.1 K11H3.1	0.00	0.27	0.00	0.09	0.00	0.44	0.00	0.15	1.66
Q9U2A8	rpl-43 rpl-37a Y48B6A.2	0.25	0.27	0.28	0.26	0.44	0.44	0.43	0.44	1.66
Q23312	rps-7 ZC434.2	0.28	0.26	0.29	0.28	0.46	0.46	0.46	0.46	1.66
Q27488	pas-2 D1054.2	0.28	0.00	0.27	0.18	0.48	0.00	0.45	0.31	1.66
P34574	chc-1 T20G5.1	0.27	0.35	0.29	0.31	0.52	0.45	0.56	0.51	1.66
Q10121	C23G10.2	0.29	0.28	0.27	0.28	0.46	0.46	0.48	0.47	1.66
O44411	nog-1 T07A9.9	0.00	0.29	0.00	0.10	0.00	0.48	0.00	0.16	1.66
P48154	rps-1 F56F3.5	0.28	0.28	0.28	0.28	0.47	0.47	0.46	0.46	1.67
O45525	CELE_F45H10.2 F45H10.2	0.27	0.28	0.28	0.28	0.46	0.45	0.48	0.46	1.67
Q20865	CELE_F56C9.7 F56C9.7	0.31	0.32	0.30	0.31	0.48	0.62	0.44	0.51	1.67
Q17770	pdi-2 C07A12.4	0.28	0.27	0.29	0.28	0.47	0.47	0.47	0.47	1.67
G5EBI0	4D656 CELE_Y54G2A.18 Y54G2A.18	0.30	0.27	0.26	0.28	0.48	0.47	0.44	0.46	1.67
P91133	ubr-1 C32E8.11	0.22	0.00	0.00	0.07	0.37	0.00	0.00	0.12	1.67
Q23050	clik-1 CELE_T25F10.6 T25F10.6	0.28	0.26	0.29	0.27	0.45	0.46	0.45	0.46	1.67
O18687	CELE_F26E4.6 F26E4.6	0.28	0.00	0.00	0.09	0.46	0.00	0.00	0.15	1.67
O16259	sti-1 R09E12.3	0.28	0.29	0.29	0.29	0.48	0.49	0.46	0.48	1.67
G5EC98	ctps-1 CELE_W06H3.3 W06H3.3	0.00	0.27	0.28	0.18	0.00	0.46	0.46	0.31	1.67
P43509	cpr-5 W07B8.5	0.30	0.28	0.24	0.27	0.39	0.46	0.53	0.46	1.67
P46562	alh-9 F01F1.6	0.28	0.28	0.28	0.28	0.45	0.47	0.46	0.46	1.68
Q9TZQ3	pgl-1 ZK381.4	0.26	0.28	0.30	0.28	0.47	0.47	0.47	0.47	1.68
Q18066	dim-1 C18A11.7/C18A11.8	0.28	0.29	0.29	0.29	0.47	0.49	0.47	0.48	1.68
O17953	dld-1 LLC1.3	0.28	0.29	0.28	0.28	0.46	0.49	0.48	0.47	1.68

P50305	sams-3 C06E7.1	0.28	0.28	0.28	0.28	0.47	0.47	0.47	0.47	1.68
Q18115	rpn-2 C23G10.4	0.27	0.29	0.32	0.30	0.54	0.48	0.47	0.50	1.68
O02056	rpl-4 B0041.4	0.30	0.28	0.26	0.28	0.47	0.47	0.47	0.47	1.68
O18236	nuo-3 CELE_Y57G11C.12 Y57G11C.12	0.28	0.29	0.27	0.28	0.46	0.48	0.48	0.47	1.68
Q9BL19	rpl-17 Y48G8AL.8	0.28	0.26	0.27	0.27	0.44	0.45	0.46	0.45	1.69
Q20219	irg-7 drd-2 CELE_F40F4.6 F40F4.6	0.27	0.28	0.29	0.28	0.46	0.47	0.48	0.47	1.69
Q9XW16	pfn-1 Y18D10A.20	0.30	0.30	0.29	0.30	0.47	0.41	0.64	0.50	1.69
Q22053	fib-1 T01C3.7	0.26	0.28	0.28	0.28	0.51	0.45	0.44	0.46	1.69
Q09607	gst-36 R07B1.4	0.29	0.00	0.29	0.19	0.48	0.00	0.50	0.33	1.69
O45934	CELE_Y43F4B.5 Y43F4B.5	0.29	0.00	0.00	0.10	0.50	0.00	0.00	0.17	1.69
Q22054	rps-16 T01C3.6	0.29	0.30	0.27	0.28	0.46	0.48	0.50	0.48	1.69
Q93235	nkb-1 C17E4.9	0.29	0.28	0.35	0.31	0.46	0.47	0.63	0.52	1.69
G5EF37	pat-10 tnc-1 CELE_F54C1.7 F54C1.7	0.28	0.45	0.29	0.34	0.53	0.61	0.57	0.57	1.69
O76371	rpt-5 CELE_F56H1.4 F56H1.4	0.40	0.28	0.29	0.32	0.61	0.46	0.58	0.55	1.69
P54688	bcat-1 eca-39 eca39 K02A4.1	0.29	0.26	0.30	0.28	0.46	0.47	0.52	0.48	1.70
O01925	mboa-6 R155.1	0.00	0.30	0.00	0.10	0.00	0.51	0.00	0.17	1.70
Q11067	tag-320 B0403.4	0.29	0.28	0.27	0.28	0.47	0.49	0.47	0.48	1.70
P90921	asg-1 K07A12.3	0.00	0.26	0.00	0.09	0.00	0.43	0.00	0.14	1.70
Q9N3D0	CELE_Y54E10BR.5 Y54E10BR.5	0.00	0.00	0.27	0.09	0.00	0.00	0.46	0.15	1.70
Q21276	K07C5.4	0.29	0.26	0.28	0.28	0.46	0.49	0.46	0.47	1.70
Q9N4X8	gst-10 Y45G12C.2	0.30	0.31	0.27	0.29	0.50	0.47	0.52	0.50	1.70
P48158	rpl-23 B0336.10	0.32	0.26	0.25	0.28	0.46	0.45	0.50	0.47	1.71
Q19102	ard-1 CELE_F01G4.2 F01G4.2	0.28	0.29	0.25	0.27	0.51	0.47	0.43	0.47	1.71
Q22067	got-1.2 T01C8.5	0.00	0.28	0.32	0.20	0.00	0.52	0.50	0.34	1.71
P91249	col-20 CELE_F11G11.11 F11G11.11	0.26	0.00	0.28	0.18	0.46	0.00	0.47	0.31	1.71
Q86LS4	dao-2 CELE_M03A1.7 M03A1.7	0.00	0.27	0.25	0.17	0.00	0.41	0.49	0.30	1.71
Q18359	C33A12.1	0.25	0.28	0.24	0.26	0.45	0.43	0.46	0.44	1.71
P34528	K12H4.7	0.30	0.29	0.23	0.27	0.49	0.40	0.51	0.47	1.71
Q86MI3	CELE_Y71H10B.1 Y71H10B.1	0.29	0.00	0.31	0.20	0.50	0.00	0.52	0.34	1.72
Q9N3T5	spg-7 CELE_Y47G6A.10 Y47G6A.10	0.30	0.24	0.00	0.18	0.45	0.47	0.00	0.31	1.72
Q18786	snr-4 C52E4.3	0.28	0.00	0.00	0.09	0.48	0.00	0.00	0.16	1.72
P41988	cct-1 tcp-1 T05C12.7	0.29	0.27	0.25	0.27	0.47	0.45	0.47	0.47	1.72
Q17405	AC3.5	0.00	0.30	0.00	0.10	0.00	0.51	0.00	0.17	1.73
Q19973	cec-5 CELE_F32E10.6 F32E10.6	0.26	0.00	0.00	0.09	0.44	0.00	0.00	0.15	1.73
P34629	lap-1 ZK353.6	0.22	0.00	0.00	0.07	0.38	0.00	0.00	0.13	1.73
O16785	pat-6 T21D12.4	0.00	0.00	0.26	0.09	0.00	0.00	0.46	0.15	1.73
Q9XUB7	far-6 CELE_W02A2.2 W02A2.2	0.26	0.26	0.30	0.27	0.46	0.45	0.48	0.47	1.73
Q9XVF7	rpl-8 rpl-2 B0250.1	0.28	0.27	0.28	0.28	0.49	0.46	0.48	0.48	1.73
G5EBK3	erm-1 C01G8.5 CELE_C01G8.5	0.23	0.32	0.30	0.28	0.53	0.49	0.45	0.49	1.73
H2FLL1	lea-1 CELE_K08H10.1 K08H10.1	0.30	0.28	0.27	0.28	0.50	0.47	0.50	0.49	1.73
Q6A8K1	act-4 CELE_M03F4.2 M03F4.2	0.23	0.29	0.26	0.26	0.46	0.43	0.46	0.45	1.73
Q93791	**nid-1 CELE_F54F3.1 F54F3.1**	0.24	0.00	0.00	0.08	0.41	0.00	0.00	0.14	1.73
O44887	inx-13 inx CELE_Y8G1A.2 Y8G1A.2	0.24	0.00	0.00	0.08	0.42	0.00	0.00	0.14	1.73
Q9U1W1	tfg-1 CELE_Y63D3A.5 Y63D3A.5	0.26	0.27	0.30	0.27	0.50	0.47	0.46	0.48	1.73
G5ECC3	rme-1 CELE_W06H8.1 W06H8.1	0.00	0.36	0.00	0.12	0.00	0.63	0.00	0.21	1.74
O18178	pptr-1 W08G11.4	0.30	0.00	0.35	0.22	0.55	0.00	0.57	0.37	1.74
Q09249	C16C10.3	0.00	0.00	0.27	0.09	0.00	0.00	0.47	0.16	1.74
O02328	eif-3.C T23D8.4	0.24	0.30	0.28	0.27	0.50	0.44	0.49	0.47	1.74

P62784	his-1 T10C6.14; his-5 F45F2.3; his-10 ZK131.4; his-14 ZK131.8; his-18 K06C4.10; his-26 ZK131.1; his-28 K06C4.2; his-31 F17E9.12; his-37 C50F4.7; his-38 K03A1.6; his-46 B0035.9; his-50 F07B7.9; his-56 F54E12.3; his-60 F55G1.11; his-64 F22B3.1; his-67 T23D8.5	0.29	0.26	0.28	0.27	0.48	0.48	0.48	0.48	1.74
Q9N350	CELE_Y55F3BR.6 Y55F3BR.6	0.24	0.32	0.25	0.27	0.48	0.45	0.47	0.47	1.74
G5EFL5	alp-1 CELE_T11B7.4 T11B7.4	0.00	0.27	0.35	0.21	0.00	0.57	0.52	0.36	1.75
Q966I8	pbs-1 CELE_K08D12.1 K08D12.1	0.25	0.24	0.31	0.27	0.49	0.46	0.45	0.47	1.75
Q10943	arf-1.2 arf-1 B0336.2	0.29	0.28	0.26	0.28	0.47	0.48	0.50	0.48	1.75
Q9N4Y8	nuo-5 CELE_Y45G12B.1 Y45G12B.1	0.29	0.22	0.32	0.28	0.47	0.53	0.44	0.48	1.75
Q95Y72	dss-1 Y119D3B.15	0.00	0.00	0.30	0.10	0.00	0.00	0.52	0.17	1.75
O16520	erfa-1 etf-1 T05H4.6	0.00	0.00	0.36	0.12	0.00	0.00	0.64	0.21	1.75
P46550	cct-6 F01F1.8	0.27	0.27	0.27	0.27	0.48	0.46	0.48	0.48	1.76
P18334	kin-3 B0205.7	0.26	0.26	0.28	0.27	0.48	0.47	0.45	0.47	1.76
P52872	dad-1 F57B10.10	0.00	0.28	0.00	0.09	0.00	0.48	0.00	0.16	1.76
Q20641	nmy-1 CELE_F52B10.1 F52B10.1	0.28	0.00	0.27	0.18	0.45	0.00	0.51	0.32	1.76
Q21888	CELE_R102.2 R102.2	0.00	0.24	0.00	0.08	0.00	0.42	0.00	0.14	1.76
Q20034	CELE_F35D11.4 F35D11.4	0.23	0.00	0.31	0.18	0.48	0.00	0.48	0.32	1.76
Q20751	iff-2 F54C9.1	0.25	0.28	0.29	0.27	0.54	0.47	0.45	0.48	1.77
P47209	cct-5 C07G2.3	0.26	0.30	0.28	0.28	0.51	0.48	0.50	0.50	1.77
Q27511	htz-1 R08C7.3	0.00	0.30	0.00	0.10	0.00	0.52	0.00	0.17	1.77
O45181	CELE_K07H8.10 K07H8.10	0.21	0.28	0.29	0.26	0.52	0.43	0.44	0.46	1.77
O62106	eif-6 C47B2.5	0.27	0.22	0.00	0.16	0.40	0.47	0.00	0.29	1.77
Q9XWJ5	CELE_Y51H1A.3 Y51H1A.3	0.26	0.00	0.27	0.18	0.47	0.00	0.46	0.31	1.78
G4SF79	CELE_F25E2.2 F25E2.2	0.00	0.00	0.26	0.09	0.00	0.00	0.47	0.16	1.78
P34652	cnx-1 ZK632.6	0.27	0.24	0.39	0.30	0.54	0.46	0.61	0.54	1.78
Q9BMU4	atln-1 4D561 CELE_Y54G2A.2 Y54G2A.2	0.00	0.36	0.00	0.12	0.00	0.64	0.00	0.21	1.79
Q1XFY9	rps-24 CELE_T07A9.11 T07A9.11	0.26	0.30	0.29	0.28	0.57	0.47	0.47	0.51	1.79
A3QMC5	rpl-34 C42C1.14 CELE_C42C1.14	0.25	0.24	0.28	0.26	0.47	0.47	0.45	0.46	1.80
Q09456	col-80 C09G5.5	0.26	0.23	0.00	0.16	0.44	0.45	0.00	0.30	1.80
Q9U3Q6	ugt-22 C08F11.8 CELE_C08F11.8	0.00	0.00	0.24	0.08	0.00	0.00	0.43	0.14	1.80
Q22253	rpn-9 CELE_T06D8.8 T06D8.8	0.27	0.00	0.00	0.09	0.48	0.00	0.00	0.16	1.80
G5EDI2	ret-1 CELE_W06A7.3 W06A7.3	0.27	0.25	0.29	0.27	0.47	0.50	0.50	0.49	1.80
P17140	**let-2 clb-1 F01G12.5**	0.24	0.00	0.26	0.17	0.45	0.00	0.45	0.30	1.80
Q11117	lmp-1 C03B1.12	0.00	0.00	0.29	0.10	0.00	0.00	0.52	0.17	1.80
P52821	rps-25 K02B2.5	0.29	0.25	0.25	0.26	0.47	0.47	0.48	0.47	1.81
Q06561	unc-52 ZC101.2	0.24	0.29	0.24	0.25	0.49	0.46	0.44	0.46	1.81
O61742	rpn-10 B0205.3	0.00	0.00	0.27	0.09	0.00	0.00	0.49	0.16	1.81
Q9XUV0	pbs-5 CELE_K05C4.1 K05C4.1	0.23	0.29	0.29	0.27	0.55	0.50	0.42	0.49	1.81
G5EDQ2	hmg-12 hmg-I-beta CELE_Y17G7A.1 Y17G7A.1	0.25	0.25	0.28	0.26	0.50	0.46	0.47	0.48	1.82
P91017	hpo-32 C01G8.6 CELE_C01G8.6	0.31	0.00	0.32	0.21	0.48	0.00	0.68	0.38	1.82
Q09510	mlc-4 C56G7.1	0.25	0.00	0.00	0.08	0.46	0.00	0.00	0.15	1.82
Q8TA47	CELE_Y51F10.7 Y51F10.7	0.00	0.00	0.35	0.12	0.00	0.00	0.65	0.22	1.82
P53703	cchl-1 T06D8.6	0.22	0.00	0.00	0.07	0.40	0.00	0.00	0.13	1.83
Q9N362	CELE_Y55F3AM.13 Y55F3AM.13	0.29	0.28	0.00	0.19	0.57	0.46	0.00	0.34	1.83
O44782	vpr-1 CELE_F33D11.11 F33D11.11	0.00	0.20	0.00	0.07	0.00	0.37	0.00	0.12	1.83
Q19437	upb-1 CELE_F13H8.7 F13H8.7	0.00	0.21	0.27	0.16	0.00	0.45	0.42	0.29	1.83
Q19162	rpl-11.2 F07D10.1	0.28	0.25	0.26	0.26	0.49	0.49	0.47	0.48	1.84
Q23058	ssp-9 E03H12.10; ssp-11 T28H11.6	0.25	0.21	0.28	0.25	0.45	0.48	0.44	0.46	1.84
Q95Y29	CELE_Y34B4A.6 Y34B4A.6	0.28	0.26	0.21	0.25	0.44	0.47	0.48	0.46	1.84
Q8MQC6	cpr-6 C25B8.3 CELE_C25B8.3	0.27	0.27	0.27	0.27	0.52	0.46	0.51	0.50	1.85

Q9TXP0	rps-27 F56E10.4	0.28	0.25	0.26	0.26	0.49	0.49	0.47	0.48	1.85
Q9XWD1	acs-5 CELE_Y76A2B.3 Y76A2B.3	0.26	0.25	0.00	0.17	0.48	0.47	0.00	0.32	1.85
O45502	dnj-12 CELE_F39B2.10 F39B2.10	0.31	0.00	0.29	0.20	0.60	0.00	0.51	0.37	1.85
Q21086	nst-1 K01C8.9	0.00	0.21	0.00	0.07	0.00	0.38	0.00	0.13	1.85
Q2PJ97	CELE_F52E1.14 F52E1.14	0.26	0.27	0.21	0.25	0.46	0.43	0.49	0.46	1.86
Q18947	ule-3 CELE_D1054.11 D1054.11	0.00	0.00	0.17	0.06	0.00	0.00	0.31	0.10	1.86
P36609	ncs-2 F10G8.5	0.29	0.32	0.28	0.30	0.56	0.49	0.59	0.55	1.86
Q6AHR3	CELE_R151.2 R151.2	0.19	0.31	0.26	0.25	0.49	0.46	0.45	0.47	1.86
Q09657	CELE_ZK1320.9 ZK1320.9	0.00	0.25	0.00	0.08	0.00	0.47	0.00	0.16	1.87
Q967F1	eif-1 CELE_T27F7.3 T27F7.3	0.26	0.00	0.25	0.17	0.53	0.00	0.43	0.32	1.87
P45971	ostb-1 T09A5.11	0.00	0.26	0.00	0.09	0.00	0.49	0.00	0.16	1.87
Q21966	asp-4 CELE_R12H7.2 R12H7.2	0.29	0.23	0.27	0.26	0.48	0.50	0.49	0.49	1.88
Q9XUY5	nkb-3 F55F3.3	0.25	0.28	0.31	0.28	0.54	0.48	0.56	0.52	1.88
Q9BKQ9	cisd-3.2 CELE_Y67D2.3 Y67D2.3	0.33	0.31	0.24	0.29	0.68	0.48	0.48	0.54	1.88
Q564Q1	gale-1 C47B2.6 CELE_C47B2.6	0.24	0.00	0.23	0.16	0.44	0.00	0.45	0.30	1.90
O17570	rpl-38 C06B8.8	0.18	0.26	0.36	0.27	0.56	0.47	0.50	0.51	1.90
Q8MXI1	ucr-11 CELE_F57B10.14 F57B10.14	0.27	0.21	0.28	0.25	0.49	0.49	0.47	0.48	1.91
Q9XWL1	ttr-17 CELE_Y5F2A.2 Y5F2A.2	0.55	0.00	0.24	0.26	0.44	0.61	0.44	0.50	1.91
O01685	C32F10.8 CELE_C32F10.8	0.26	0.00	0.25	0.17	0.49	0.00	0.47	0.32	1.92
P55216	cth-2 ZK1127.10	0.25	0.32	0.24	0.27	0.51	0.46	0.62	0.53	1.93
C6KRN1	sao-1 R10D12.14	0.26	0.27	0.26	0.26	0.50	0.49	0.53	0.51	1.93
Q23571	dbt-1 ZK669.4	0.25	0.25	0.23	0.24	0.49	0.44	0.47	0.46	1.93
Q21568	M28.5	0.23	0.24	0.26	0.24	0.48	0.46	0.47	0.47	1.93
O17954	CELE_LLC1.2 LLC1.2	0.00	0.22	0.00	0.07	0.00	0.43	0.00	0.14	1.94
O45106	ech-5 CELE_F56B3.5 F56B3.5	0.00	0.27	0.26	0.18	0.00	0.45	0.57	0.34	1.94
Q17348	snr-1 Y116A8C.42	0.24	0.25	0.24	0.24	0.49	0.48	0.46	0.47	1.94
Q65ZJ7	C32D5.8 CELE_C32D5.8	0.26	0.00	0.24	0.17	0.46	0.00	0.51	0.33	1.94
Q23635	CELE_ZK84.1 ZK84.1	0.00	0.00	0.23	0.08	0.00	0.00	0.45	0.15	1.95
Q9U2K8	abce-1 CELE_Y39E4B.1 Y39E4B.1	0.30	0.24	0.23	0.26	0.48	0.54	0.48	0.50	1.95
Q6AW03	CELE_Y37E3.17 Y37E3.17	0.32	0.24	0.33	0.29	0.67	0.40	0.66	0.57	1.95
Q9U238	trap-4 CELE_Y56A3A.21 Y56A3A.21	0.25	0.24	0.26	0.25	0.51	0.49	0.48	0.49	1.97
P19626	mlc-2 C36E6.5	0.24	0.26	0.27	0.26	0.52	0.51	0.49	0.51	1.99
P34714	ost-1 sparc C44B12.2	0.28	0.23	0.00	0.17	0.53	0.49	0.00	0.34	2.00
G5EDY2	wars-1 wrs-1 CELE_Y80D3A.1 Y80D3A.1	0.18	0.20	0.20	0.19	0.39	0.40	0.38	0.39	2.03
O61792	rpn-8 CELE_R12E2.3 R12E2.3	0.00	0.27	0.30	0.19	0.00	0.64	0.51	0.38	2.04
O16521	hpo-19 CELE_T05H4.5 T05H4.5	0.26	0.00	0.00	0.09	0.54	0.00	0.00	0.18	2.06
P90789	D2030.4	0.00	0.21	0.21	0.14	0.00	0.44	0.46	0.30	2.13
A8WHP8	plin-1 mdt-28 CELE_W01A8.1 W01A8.1	0.00	0.23	0.00	0.08	0.00	0.49	0.00	0.16	2.13
Q20589	CELE_F49C12.12 F49C12.12	0.00	0.21	0.00	0.07	0.00	0.45	0.00	0.15	2.15
O18180	mrpl-12 CELE_W09D10.3 W09D10.3	0.27	0.27	0.18	0.24	0.54	0.49	0.53	0.52	2.15
Q09236	copd-1 C13B9.3	0.28	0.24	0.00	0.17	0.72	0.40	0.00	0.37	2.15
Q03206	ced-10 rac-1 C09G12.8	0.26	0.22	0.00	0.16	0.52	0.52	0.00	0.35	2.17
O44781	CELE_F33D11.10 F33D11.10	0.00	0.33	0.29	0.21	0.60	0.38	0.42	0.47	2.25
Q9XWV2	Y37D8A.2	0.00	0.00	0.21	0.07	0.00	0.00	0.47	0.16	2.26
Q22135	CELE_T04A8.6 T04A8.6	0.20	0.00	0.00	0.07	0.46	0.00	0.00	0.15	2.32
B7FAR9	CELE_Y43F8B.1 Y43F8B.1	0.00	0.22	0.17	0.13	0.00	0.42	0.50	0.31	2.33
O61709	CELE_R119.3 R119.3	0.00	0.21	0.00	0.07	0.00	0.50	0.00	0.17	2.34
Q9BL46	CELE_Y71H2AM.11 Y71H2AM.11	0.26	0.26	0.19	0.23	0.52	0.52	0.59	0.54	2.35
P52715	F13D12.6	0.27	0.28	0.00	0.18	0.56	0.72	0.00	0.43	2.36
G5EFV4	hmg-1.1 CELE_Y48B6A.14 Y48B6A.14	0.23	0.00	0.00	0.08	0.55	0.00	0.00	0.18	2.39

Q95YA9	cas-1 CELE_F41G4.2 F41G4.2	0.00	0.21	0.00	0.07	0.00	0.51	0.00	0.17	2.39
Q69ZI6	CELE_T12D8.10 T12D8.10	0.25	0.00	0.22	0.16	0.54	0.00	0.59	0.38	2.39
Q9XTU6	snr-6 Y49E10.15	0.19	0.00	0.00	0.06	0.46	0.00	0.00	0.15	2.41
P34255	B0303.3	0.31	0.00	0.31	0.20	0.51	0.59	0.41	0.51	2.48
Q8I711	gars-1 CELE_T10F2.1 T10F2.1	0.23	0.00	0.00	0.08	0.59	0.00	0.00	0.20	2.53
O16368	rpt-2 F29G9.5	0.35	0.00	0.23	0.19	0.43	0.55	0.49	0.49	2.54
G2HK03	pqn-87 CELE_Y57A10A.18 Y57A10A.18	0.41	0.00	0.00	0.14	0.41	0.00	0.65	0.35	2.54
Q09545	sdhb-1 tag-55 F42A8.2	0.33	0.33	0.00	0.22	0.57	0.41	0.80	0.59	2.70
G5EG85	unc-70 bgs-1 CELE_K11C4.3 K11C4.3	0.00	0.41	0.00	0.14	0.83	0.33	0.00	0.39	2.81
O17352	aagr-4 CELE_F52D1.1 F52D1.1	0.00	0.00	0.31	0.10	0.36	0.00	0.50	0.29	2.82
O62415	lys-1 CELE_Y22F5A.4 Y22F5A.4	0.00	0.16	0.23	0.13	0.00	0.62	0.49	0.37	2.88
Q9N4L8	lpd-5 CELE_ZK973.10 ZK973.10	0.24	0.28	0.00	0.17	0.45	0.43	0.64	0.51	2.91
O02252	pcp-3 CELE_F23B2.11 F23B2.11	0.12	0.00	0.00	0.04	0.36	0.00	0.00	0.12	2.94
Q22719	CELE_T24B8.3 T24B8.3	0.00	0.33	0.30	0.21	0.71	0.58	0.60	0.63	3.00
A7DTF0	mrg-1 CELE_Y37D8A.9 Y37D8A.9	0.00	0.25	0.00	0.08	0.00	0.75	0.00	0.25	3.03
Q23487	CELE_ZK418.9 ZK418.9	0.00	0.25	0.00	0.08	0.00	0.75	0.00	0.25	3.07
H2L0M0	pod-2 CELE_W09B6.1 W09B6.1	0.00	0.28	0.00	0.09	0.00	0.85	0.00	0.28	3.08
Q10661	dpy-30 ZK863.6	0.15	0.00	0.00	0.05	0.45	0.00	0.00	0.15	3.12
C7FZU5	C41G7.9 CELE_C41G7.9	0.00	0.00	0.33	0.11	0.00	0.62	0.42	0.35	3.17
P91250	col-73 CELE_F11G11.12 F11G11.12	0.00	0.21	0.20	0.13	0.43	0.42	0.44	0.43	3.20
Q22170	ile-2 CELE_T04G9.3 T04G9.3	0.00	0.38	0.00	0.13	0.68	0.62	0.00	0.43	3.39
G5EGH7	lon-8 Y59A8B.20	0.17	0.00	0.00	0.06	0.61	0.00	0.00	0.20	3.63
G5EE74	nud-1 CELE_F53A2.4 F53A2.4	0.00	0.00	0.31	0.10	0.77	0.00	0.40	0.39	3.75
O45864	CELE_T27E9.2 T27E9.2	0.00	0.00	0.26	0.09	0.61	0.00	0.38	0.33	3.76
Q9XWM1	oig-2 CELE_Y38F1A.9 Y38F1A.9	0.21	0.00	0.19	0.13	0.44	0.59	0.50	0.51	3.77
Q21313	epi-1 K08C7.3	0.25	0.21	0.00	0.15	0.46	0.67	0.60	0.58	3.82
Q23089	xpo-1 CELE_ZK742.1 ZK742.1	0.00	0.00	0.12	0.04	0.00	0.00	0.45	0.15	3.86
O62432	dpy-4 CELE_Y41E3.2 Y41E3.2	0.07	0.16	0.13	0.12	0.50	0.47	0.44	0.47	3.98
O17287	tomm-22 W10D9.5	0.00	0.14	0.00	0.05	0.00	0.55	0.00	0.18	4.06
Q09289	C56G2.7	0.00	0.24	0.00	0.08	0.00	0.51	0.49	0.33	4.12
Q9XTT4	ani-1 Y49E10.19	0.00	0.07	0.00	0.02	0.00	0.30	0.00	0.10	4.27
H2KYI5	C06G3.5 CELE_C06G3.5	0.24	0.00	0.00	0.08	0.48	0.00	0.56	0.35	4.33
H2L076	ketn-1 CELE_F54E2.3 F54E2.3	0.00	0.00	0.25	0.08	0.00	0.59	0.51	0.37	4.45
Q93896	mct-3 CELE_M03B6.2 M03B6.2	0.00	0.32	0.00	0.11	1.00	0.42	0.00	0.47	4.51
P34183	hrs-1 syh-1 T11G6.1	0.39	0.00	0.00	0.13	0.49	0.63	0.68	0.60	4.58
Q8T3G5	egl-30 CELE_M01D7.7 M01D7.7	0.00	0.12	0.00	0.04	0.00	0.56	0.00	0.19	4.59
Q9BL07	Y54F10AM.8	0.00	0.17	0.00	0.06	0.00	0.51	0.43	0.31	5.47
Q93618	F27D4.4	0.00	0.30	0.00	0.10	0.67	0.45	0.63	0.58	5.89
O02153	grd-14 CELE_T01B10.2 T01B10.2	0.00	0.00	0.13	0.04	0.45	0.00	0.40	0.28	6.44
Q22192	ttr-14 CELE_T05A10.3 T05A10.3	0.00	0.00	0.16	0.05	0.68	0.00	0.50	0.40	7.56
Q94360	nduf-7 W10D5.2	0.08	0.00	0.00	0.03	0.57	0.00	0.61	0.39	15.57
