# Supplementary material for: A muscle-epidermis-glia signaling axis sustains synaptic specificity during allometric growth in Caenorhabditis elegans
Source: eLife. 2020 Apr 7;9:e55890. doi: 10.7554/eLife.55890 (PMC7164957; doi:10.7554/eLife.55890)
Supplement: Supplementary file 2. [file elife-55890-supp2.pdf]

Table S2. Strains used in this study	
**Strain name**	**Genotype**
N2	Wild type
TV392	*wyIs45 [Pttx-3::GFP::rab-3; Punc-122::RFP]* X
FDU937	*cima-1(wy84)* IV; *wyIs45 [Pttx-3::GFP::rab-3; Punc-122::RFP]* X
FDU2215	*shcEx1126[Pttx-3::syd-1::GFP;Pttx-3::rab-3::mCherry;Punc-122::RFP]*
FDU2216	*cima-1(wy84)* IV;*shcEx1127[Pttx-3::syd-1::GFP;Pttx-3::rab-3::mCherry;Punc-122::RFP]*
FDU2217	*cima-1(wy84)* IV;*mig-17(ols226)* V;*shcEx1128[Pttx-3::syd-1::GFP;Pttx-3::rab-3::mCherry;Punc-122::RFP]*
FDU1140	*shcEx776 [Phlh-17::mCherry; Pttx-3::GFP::rab-3]*
FDU1784	*cima-1(wy84)* IV; *shcEx777 [Phlh-17::mCherry; Pttx-3::GFP::rab-3]*
FDU1785	*mig-17(ola226)* V; *shcEx778 [Phlh-17::mCherry; Pttx-3::GFP::rab-3]*
FDU1143	*cima-1(wy84)* IV; *mig-17(ola226)* V; *shcEx780 [Phlh-17::mCherry; Pttx-3::GFP::rab-3]*
FDU2095	*cima-1(wy84)* IV; *mig-17(ola226)* V; *wyIs45 [Pttx-3::GFP::rab-3; Punc-122::RFP]* X
FDU2333	*cima-1(wy84)* IV; *mig-17(ola226)* V; *wyIs45 [Pttx-3::GFP::rab-3; Punc-122::RFP]* X; *shcEx1146 [Pmig-17::mig-17 genomics; Phlh-17::mCherry]*
FDU2334	*cima-1(wy84)* IV; *mig-17(ola226)* V; *wyIs45 [Pttx-3::GFP::rab-3; Punc-122::RFP]* X; *shcEx1147 [Pmig-17::mig-17 genomics; Phlh-17::mCherry]*
FDU882	*cima-1(wy84)* IV; *mig-17(k113)* V; *wyIs45 [Pttx-3::GFP::rab-3; Punc-122::RFP]* X
FDU2315	*cima-1(wy84)* IV; *mig-17(k174)* V; *wyIs45 [Pttx-3::GFP::rab-3; Punc-122::RFP]* X
FDU2219	*shcEx1129[Pmig-17::mig-17::SL2::GFP; Pdpy-4::mCherry]*
FDU2220	*shcEx1130[Pmig-17::mig-17::SL2::GFP; Pmyo-3::mCherry]*
FDU2318	*shcEx1131[Pmig-17::mig-17::SL2::GFP; Phlh-17::mCherry]*
FDU2462	*shcEx1410[Pmig-17::mig-17::SL2::GFP; Prab-3::mCherry]*
FDU2214	*cima-1(wy84)* IV; *mig-17(shc8)* V; *wyIs45 [Pttx-3::GFP::rab-3; Punc-122::RFP]* X
FDU2596	*cima-1(wy84)* IV; *mig-17(ola226)* V; *wyIs45 [Pttx-3::GFP::rab-3; Punc-122::RFP]* X; *shcEx1414 [Pmig-17::mig-17(E303A); Phlh-17::mCherry]*
FDU2597	*cima-1(wy84)* IV; *mig-17(ola226)* V; *wyIs45 [Pttx-3::GFP::rab-3; Punc-122::RFP]* X; *shcEx 1415[Pmig-17::mig-17(E303A); Phlh-17::mCherry]*
FDU3202	*cima-1(wy84)* IV; *mig-17(ola226)* V; *wyIs45 [Pttx-3::GFP::rab-3; Punc-122::RFP]* X; *shcEx1682[Prab-3::mig-17; Phlh-17::mCherry]*
FDU3203	*cima-1(wy84)* IV; *mig-17(ola226)* V; *wyIs45 [Pttx-3::GFP::rab-3; Punc-122::RFP]* X; *shcEx1683[Prab-3::mig-17; Phlh-17::mCherry]*
FDU1056	*mig-17(shc19)* V
FDU1778	*mig-17(shc19)* V; *shcEx845[Phlh-17::mCherry]*
FDU2332	*mig-17(shc19)* V; *shcEx1145[Pdpy-4::mCherry]*
FDU1896	*mig-17(shc19)* V; *shcEx1402[Pmyo-3::mCherry]*
FDU1891	*mig-17(shc19)* V;*shcEx1403[Prab-3::mCherry]*
FDU1518	*cima-1(wy84)* IV; *emb-9(xd51)* III; *wyIs45 [Pttx-3::GFP::rab-3; Punc-122::RFP]* X

FDU192	*cima-1(wy84)* IV*; emb-9(tk75)* III*; wyIs45 [Pttx-3::GFP::rab-3; Punc-122::RFP]* X
FDU2945	*cima-1(wy84)* IV*; emb-9(b189)* IV*;wyIs45 [Pttx-3::GFP::rab-3; Punc-122::RFP]* X
DCR1475	*cima-1(wy84)* IV*; unc-52(e1421)* II*; wyIs45[Pttx-3::GFP::rab-3; Punc-122::RFP]* X
FDU38	*cima-1(wy84)* IV*; fbl-1(k201)* IV*; wyIs45;[Pttx-3::GFP::rab-3; Punc-122::RFP]* X
FDU45	*cima-1(wy84)* IV*; mig-17(ola226)* V*; fbl-1(k201)* IV*; wyIs45 [Pttx-3::GFP::rab-3; Punc-122::RFP]* X
FDU2598	*mig-17(shc19)* V*; qyIs46[unc119;emb-9::mCherry]* X
NK364	*unc-119(ed4)* III*; qyIs46[unc119;emb-9::mCherry]* X
FDU2593	*cima-1(wy84)* IV*; qyIs46[unc119;emb-9::mCherry]* X
FDU2594	*mig-17(ola226)* V*;qyIs46[unc119;emb-9::mCherry]* X
FDU2595	*cima-1(wy84)* IV*; mig-17(ola226)* V*; qyIs46[unc119;emb-9::mCherry]* X
FDU2314	*mig-17(k174)V;wyIs45[Pttx-3::GFP::rab-3; Punc-122::RFP]* X
FDU883	*mig-17(k113)* V*; wyIs45 [Pttx-3::GFP::rab-3; Punc-122::RFP]* X
FDU2583	*wyIs45[Pttx-3::GFP::rab-3; Punc-122::RFP]* X*; shcEx1252[Pmig-17::mig-17 genomics; Phlh-17::mCherry]*
FDU2584	*wyIs45[Pttx-3::GFP::rab-3; Punc-122::RFP]* X*; shcEx1253[Pmig-17::mig-17 genomics; Phlh-17::mCherry]*
FDU3143	*fbl-1(k201)* IV*;wyIs45[Pttx-3::GFP::rab-3; Punc-122::RFP]* X*;shcEx1686[Phlh-17::mCherry]*
FDU3144	*fbl-1(k201)* IV*;wyIs45[Pttx-3::GFP::rab-3; Punc-122::RFP]* X*;shcEx1687[Phlh-17::mCherry]*
FDU3192	*fbl-1(k201)* IV*;wyIs45[Pttx-3::GFP::rab-3; Punc-122::RFP]* X*;shcEx1691[Phlh-17::mCherry]*
FDU3145	*let-2(b246)* X*;shcEx1688[Phlh-17::mCherry]*
FDU3146	*let-2(b246)* X*;shcEx1689[Phlh-17::mCherry]*
FDU3147	*let-2(b246)* X*;shcEx1690[Phlh-17::mCherry]*
FDU3193	*unc-52(e1421)* II*;wyIs45[Pttx-3::GFP::rab-3; Punc-122::RFP]* X*;shcEx1692[Phlh-17::mCherry]*
FDU3194	*unc-52(e1421)* II*;wyIs45[Pttx-3::GFP::rab-3; Punc-122::RFP]* X*;shcEx1693[Phlh-17::mCherry]*
FDU1519	*emb-9(xd51)* III; *wyIs45 [Pttx-3::GFP::rab-3; Punc-122::RFP]* X
FDU2995	*emb-9(b189)* IV*;wyIs45 [Pttx-3::GFP::rab-3; Punc-122::RFP]* X
DCR4032	*fbl-1(k201)* IV;*wyIs45 [Pttx-3::GFP::rab-3; Punc-122::RFP]* X
DCR1624	*unc-52(gk3)* II, *wyIs45[Pttx-3::GFP::rab-3; Punc-122::RFP]* X
FDU3101	*unc-52(e1421)* II;*wyIs45[Pttx-3::GFP::rab-3; Punc-122::RFP]* X
FDU2320	*cima-1(wy84)* IV*; mig-17(ola226)* V*; wyIs45 [Pttx-3::GFP::rab-3; Punc-122::RFP]* X*; shcEx1133 [Pmyo-3::mig-17; Phlh-17::mCherry]*
FDU2321	*cima-1(wy84)* IV*; mig-17(ola226)* V*; wyIs45 [Pttx-3::GFP::rab-3; Punc-122::RFP]* X*; shcEx1134 [Pmyo-3::mig-17; Phlh-17::mCherry]*
FDU2322	*cima-1(wy84)* IV*; mig-17(ola226)* V*; wyIs45 [Pttx-3::GFP::rab-3; Punc-122::RFP]* X*; shcEx1135 [Pmyo-3::mig-17; Phlh-17::mCherry]*
FDU2326	*cima-1(wy84)* IV*; mig-17(ola226)* V*; wyIs45 [Pttx-3::GFP::rab-3; Punc-122::RFP]* X*; shcEx1139 [Phlh-17::mig-17; Phlh-17::mCherry]*
FDU2327	*cima-1(wy84)* IV*; mig-17(ola226)* V*; wyIs45 [Pttx-3::GFP::rab-3; Punc-122::RFP]* X*; shcEx1140 [Phlh-17::mig-17; Phlh-17::mCherry]*

FDU2329	*cima-1(wy84)* IV; *mig-17(ola226)* V; *wyIs45 [Pttx-3::GFP::rab-3; Punc-122::RFP]* X; *shcEx1142 [Pdpy-7::mig-17; Phlh-17::mCherry]*
FDU2330	*cima-1(wy84)* IV; *mig-17(ola226)* V; *wyIs45 [Pttx-3::GFP::rab-3; Punc-122::RFP]* X; *shcEx1143 [Pdpy-7::mig-17; Phlh-17::mCherry]*
FDU3195	*cima-1(wy84)* IV; *mig-17(ola226)* V; *wyIs45 [Pttx-3::GFP::rab-3; Punc-122::RFP]* X; *shcEx1675[Pttx-3::mig-17; Phlh-17::mCherry]*
FDU3196	*cima-1(wy84)* IV; *mig-17(ola226)* V; *wyIs45 [Pttx-3::GFP::rab-3; Punc-122::RFP]* X; *shcEx1676[Plim-4::mig-17; Phlh-17::mCherry]*
FDU3197	*cima-1(wy84)* IV; *mig-17(ola226)* V; *wyIs45 [Pttx-3::GFP::rab-3; Punc-122::RFP]* X; *shcEx1677[Plim-4::mig-17; Phlh-17::mCherry]*
FDU3198	*cima-1(wy84)* IV; *mig-17(ola226)* V; *wyIs45 [Pttx-3::GFP::rab-3; Punc-122::RFP]* X; *shcEx1678[Plim-4::mig-17; Phlh-17::mCherry]*
FDU3199	*cima-1(wy84)* IV; *mig-17(ola226)* V; *wyIs45 [Pttx-3::GFP::rab-3; Punc-122::RFP]* X; *shcEx1679[Plim-4::mig-17; Phlh-17::mCherry]*
FDU3204	*cima-1(wy84)* IV; *mig-17(ola226)* V; *wyIs45 [Pttx-3::GFP::rab-3; Punc-122::RFP]* X; *shcEx1684[Pttx-3::mig-17; Phlh-17::mCherry]*
FDU3205	*cima-1(wy84)* IV; *mig-17(ola226)* V; *wyIs45 [Pttx-3::GFP::rab-3; Punc-122::RFP]* X; *shcEx1685[Pttx-3::mig-17; Phlh-17::mCherry]*
FDU3207	*unc-119(ed4)* III; *qyIs46[unc119;emb-9::mCherry]* X; *shcEx1695[Pmyo-3::GFP]*
FDU3208	*unc-119(ed4)* III;*qyIs46[unc119;emb-9::mCherry]* X; *shcEx1696[Pmyo-3::GFP]*
FDU3209	*unc-119(ed4)* III;*qyIs46[unc119;emb-9::mCherry]* X; *shcEx1697[Pttx-3::GFP]*
FDU3210	*unc-119(ed4)* III;*qyIs46[unc119;emb-9::mCherry]* X; *shcEx1698[Pttx-3::GFP]*
FDU3211	*unc-119(ed4)* III;*qyIs46[unc119;emb-9::mCherry]* X; *shcEx1699[Phlh-17::GFP]*
