# Supplementary material for: A muscle-epidermis-glia signaling axis sustains synaptic specificity during allometric growth in Caenorhabditis elegans
Source: eLife. 2020 Apr 7;9:e55890. doi: 10.7554/eLife.55890 (PMC7164957; doi:10.7554/eLife.55890)
Supplement: Supplementary file 4. [file elife-55890-supp4.docx]

**Supplemental file 4: Key Resources Table**

| **Key Resources Table** | | | | |
| --- | --- | --- | --- | --- |
| **Reagent type (species) or resource** | **Designation** | **Source or reference** | **Identifiers** | **Additional information** |
| genetic reagent (*C. elegans*) | N2 | (Brenner, 1974) |  |  |
| genetic reagent (*C. elegans*) | TV392 | (Colon-Ramos et al., 2007) |  |  |
| genetic reagent (*C. elegans*) | FDU937 | This study |  | Strain maintained in Shao lab |
| genetic reagent (*C. elegans*) | FDU2215 | This study |  | Strain maintained in Shao lab |
| genetic reagent (*C. elegans*) | FDU2216 | This study |  | Strain maintained in Shao lab |
| genetic reagent (*C. elegans*) | FDU2217 | This study |  | Strain maintained in Shao lab |
| genetic reagent (*C. elegans*) | FDU1140 | This study |  | Strain maintained in Shao lab |
| genetic reagent (*C. elegans*) | FDU1784 | This study |  | Strain maintained in Shao lab |
| genetic reagent (*C. elegans*) | FDU1785 | This study |  | Strain maintained in Shao lab |
| genetic reagent (*C. elegans*) | FDU1143 | This study |  | Strain maintained in Shao lab |
| genetic reagent (*C. elegans*) | FDU2095 | This study |  | Strain maintained in Shao lab |
| genetic reagent (*C. elegans*) | FDU2333 | This study |  | Strain maintained in Shao lab |
| genetic reagent (*C. elegans*) | FDU2334 | This study |  | Strain maintained in Shao lab |
| genetic reagent (*C. elegans*) | FDU882 | This study |  | Strain maintained in Shao lab |
| genetic reagent (*C. elegans*) | FDU2315 | This study |  | Strain maintained in Shao lab |
| genetic reagent (*C. elegans*) | FDU2219 | This study |  | Strain maintained in Shao lab |
| genetic reagent (*C. elegans*) | FDU2220 | This study |  | Strain maintained in Shao lab |
| genetic reagent (*C. elegans*) | FDU2318 | This study |  | Strain maintained in Shao lab |
| genetic reagent (*C. elegans*) | FDU2462 | This study |  | Strain maintained in Shao lab |
| genetic reagent (*C. elegans*) | FDU2214 | This study |  | Strain maintained in Shao lab |
| genetic reagent (*C. elegans*) | FDU2596 | This study |  | Strain maintained in Shao lab |
| genetic reagent (*C. elegans*) | FDU2597 | This study |  | Strain maintained in Shao lab |
| genetic reagent (*C. elegans*) | FDU3202 | This study |  | Strain maintained in Shao lab |
| genetic reagent (*C. elegans*) | FDU3203 | This study |  | Strain maintained in Shao lab |
| genetic reagent (*C. elegans*) | FDU1056 | This study |  | Strain maintained in Shao lab |
| genetic reagent (*C. elegans*) | FDU1778 | This study |  | Strain maintained in Shao lab |
| genetic reagent (*C. elegans*) | FDU2332 | This study |  | Strain maintained in Shao lab |
| genetic reagent (*C. elegans*) | FDU1896 | This study |  | Strain maintained in Shao lab |
| genetic reagent (*C. elegans*) | FDU1891 | This study |  | Strain maintained in Shao lab |
| genetic reagent (*C. elegans*) | FDU1518 | This study |  | Strain maintained in Shao lab |
| genetic reagent (*C. elegans*) | FDU192 | This study |  | Strain maintained in Shao lab |
| genetic reagent (*C. elegans*) | FDU2945 | This study |  | Strain maintained in Shao lab |
| genetic reagent (*C. elegans*) | DCR1475 | This study |  | Strain maintained in Colόn-Ramos lab |
| genetic reagent (*C. elegans*) | FDU38 | This study |  | Strain maintained in Shao lab |
| genetic reagent (*C. elegans*) | FDU45 | This study |  | Strain maintained in Shao lab |
| genetic reagent (*C. elegans*) | FDU2598 | This study |  | Strain maintained in Shao lab |
| genetic reagent (*C. elegans*) | NK364 | (Ihara et al., 2011) |  | A gift from Dr. DR. Sherwood |
| genetic reagent (*C. elegans*) | FDU2593 | This study |  | Strain maintained in Shao lab |
| genetic reagent (*C. elegans*) | FDU2594 | This study |  | Strain maintained in Shao lab |
| genetic reagent (*C. elegans*) | FDU2595 | This study |  | Strain maintained in Shao lab |
| genetic reagent (*C. elegans*) | FDU2314 | This study |  | Strain maintained in Shao lab |
| genetic reagent (*C. elegans*) | FDU883 | This study |  | Strain maintained in Shao lab |
| genetic reagent (*C. elegans*) | FDU2583 | This study |  | Strain maintained in Shao lab |
| genetic reagent (*C. elegans*) | FDU2584 | This study |  | Strain maintained in Shao lab |
| genetic reagent (*C. elegans*) | FDU3143 | This study |  | Strain maintained in Shao lab |
| genetic reagent (*C. elegans*) | FDU3144 | This study |  | Strain maintained in Shao lab |
| genetic reagent (*C. elegans*) | FDU3192 | This study |  | Strain maintained in Shao lab |
| genetic reagent (*C. elegans*) | FDU3145 | This study |  | Strain maintained in Shao lab |
| genetic reagent (*C. elegans*) | FDU3146 | This study |  | Strain maintained in Shao lab |
| genetic reagent (*C. elegans*) | FDU3147 | This study |  | Strain maintained in Shao lab |
| genetic reagent (*C. elegans*) | FDU3193 | This study |  | Strain maintained in Shao lab |
| genetic reagent (*C. elegans*) | FDU3194 | This study |  | Strain maintained in Shao lab |
| genetic reagent (*C. elegans*) | FDU1519 | This study |  | Strain maintained in Shao lab |
| genetic reagent (*C. elegans*) | FDU2995 | This study |  | Strain maintained in Shao lab |
| genetic reagent (*C. elegans*) | DCR4032 | This study |  | Strain maintained in Colón-Ramos lab |
| genetic reagent (*C. elegans*) | DCR1624 | This study |  | Strain maintained in Colón-Ramos lab |
| genetic reagent (*C. elegans*) | FDU3101 | This study |  | Strain maintained in Shao lab |
| genetic reagent (*C. elegans*) | FDU2320 | This study |  | Strain maintained in Shao lab |
| genetic reagent (*C. elegans*) | FDU2321 | This study |  | Strain maintained in Shao lab |
| genetic reagent (*C. elegans*) | FDU2322 | This study |  | Strain maintained in Shao lab |
| genetic reagent (*C. elegans*) | FDU2326 | This study |  | Strain maintained in Shao lab |
| genetic reagent (*C. elegans*) | FDU2327 | This study |  | Strain maintained in Shao lab |
| genetic reagent (*C. elegans*) | FDU2329 | This study |  | Strain maintained in Shao lab |
| genetic reagent (*C. elegans*) | FDU2330 | This study |  | Strain maintained in Shao lab |
| genetic reagent (*C. elegans*) | FDU3195 | This study |  | Strain maintained in Shao lab |
| genetic reagent (*C. elegans*) | FDU3196 | This study |  | Strain maintained in Shao lab |
| genetic reagent (*C. elegans*) | FDU3197 | This study |  | Strain maintained in Shao lab |
| genetic reagent (*C. elegans*) | FDU3198 | This study |  | Strain maintained in Shao lab |
| genetic reagent (*C. elegans*) | FDU3199 | This study |  | Strain maintained in Shao lab |
| genetic reagent (*C. elegans*) | FDU3204 | This study |  | Strain maintained in Shao lab |
| genetic reagent (*C. elegans*) | FDU3205 | This study |  | Strain maintained in Shao lab |
| genetic reagent (*C. elegans*) | FDU3207 | This study |  | Strain maintained in Shao lab |
| genetic reagent (*C. elegans*) | FDU3208 | This study |  | Strain maintained in Shao lab |
| genetic reagent (*C. elegans*) | FDU3209 | This study |  | Strain maintained in Shao lab |
| genetic reagent (*C. elegans*) | FDU3210 | This study |  | Strain maintained in Shao lab |
| genetic reagent (*C. elegans*) | FDU3211 | This study |  | Strain maintained in Shao lab |
| genetic reagent (*C. elegans*) | OP50 | (Brenner, 1974) |  |  |
| recombinant DNA reagent | *Pmig-17::SL2::GFP* | This study |  |  |
| recombinant DNA reagent | *Pmig-17::mig-17* | This study |  |  |
| recombinant DNA reagent | *mig-17(shc19)* Cas9-sgRNA1 | This study |  | pDD162 was described in (Dickinson et al., 2015). |
| recombinant DNA reagent | *mig-17(shc19)* Cas9-sgRNA2 | This study |  | pDD162 was described in (Dickinson et al., 2015). |
| recombinant DNA reagent | *mig-17(shc19)* repair template of CRISPR/Cas9 | This study |  | *mig-17(shc19)* contains *mig-17::mNeonGreen::3×flag.*This plasmid was constructed by enzyme digest and T4 ligation. |
| recombinant DNA reagent | *mig-17(shc8)* Cas9-sgRNA1 | This study |  | pDD162 was described in (Dickinson et al., 2015). |
| recombinant DNA reagent | *mig-17(shc8)* Cas9-sgRNA2 | This study |  | pDD162 was described in (Dickinson et al., 2015). |
| recombinant DNA reagent | *mig-17(shc8)* repair template of CRISPR/Cas9 | This study |  | *mig-17(shc8)* contains *mig-17(E303A)* where the protease catalytic site of MIG-17 is altered from E303 to A. This plasmid was constructed by enzyme digest and T4 ligation. |
| recombinant DNA reagent | *Pmig-17::mig-17(E303A)::GFP* | (Nishiwaki et al., 2000) |  | A gift from the lab of Kiyoji Nishiwaki |
| recombinant DNA reagent | *Phlh-17::mig-17* | This study |  | *Phlh-17* is published in (McMiller and Johnson, 2005). |
| recombinant DNA reagent | *Prab-3::mig-17* | This study |  | *Prab-3* was described in (Nonet et al., 1997). From the lab of Daniel A. Colón-Ramos. |
| recombinant DNA reagent | *Pdpy-7::mig-17* | This study |  | *Pdpy-7* is published in (Bulow et al., 2004). |
| recombinant DNA reagent | *Pmyo-3::mig-17* | This study |  | *Pmyo-3* was described in (Okkema et al., 1993). |
| recombinant DNA reagent | *dgn-1 RNAi* | This study |  | The full length of *dgn-1 cDNA* in L4440. |
| recombinant DNA reagent | *Pdpy-7::egl-15(5A)* | (Bulow et al., 2004) |  |  |
| recombinant DNA reagent | *Prab-3::mCherry* | *Prab-3* was described in. (Nonet et al., 1997) |  |  |
| recombinant DNA reagent | *Pttx-3g::GFP::rab-3* | (Colon-Ramos et al., 2007) |  | *Pttx-3g* was described in (Wenick and Hobert, 2004)*.* |
| recombinant DNA reagent | *Phlh-17::mCherry* | (Colon-Ramos et al., 2007) |  | *Phlh-17* is published in (McMiller and Johnson, 2005) |
| recombinant DNA reagent | *Pdpy-4::mCherry* | This study |  |  |
| chemical compound, drug | ITPG | Biosharp | Biosharp: BS044B |  |
| chemical compound, drug | Halocarbon 700 Oil | Sigma | Sigma:H8898 |  |
| chemical compound, drug | NP-40 | BBI Life Science | BBI Life Science: [A600385-0100](https://www.sangon.com/productDetail?productInfo.code=A600385) |  |
| chemical compound, drug | Tween-20 | Sigma | Sigma: P1379-500ML |  |
| chemical compound, drug | Gelatin | BBI Life Science | BBI Life Science: A609764-0100 |  |
| chemical compound, drug | PIPES | BBI Life Science | BBI Life Science: [A600719-0100](https://www.sangon.com/productDetail?productInfo.code=A600719) |  |
| chemical compound, drug | Trizol | Thermo | Thermo: 15596018 |  |
| chemical compound, drug | carbenicillin | Biosharp | Biosharp :BS042C |  |
| commercial assay or kit | RT-PCR kit | Promega | Promega:A5001 |  |
| software, algorithm | Imaris | http://www.bitplane.com/imaris/imaris | RRID:SCR_007370 |  |
| software, algorithm | GraphPad Prism | http://www.graphpad.com/ | RRID:SCR_002798 | Version :GraphPad Prism 6.01 |
| software, algorithm | Fiji | http://fiji.sc | RRID:SCR_002285 |  |

Brenner, S. (1974). The genetics of Caenorhabditis elegans. Genetics *77*, 71-94.

Bulow, H.E., Boulin, T., and Hobert, O. (2004). Differential functions of the C. elegans FGF receptor in axon outgrowth and maintenance of axon position. Neuron *42*, 367-374.

Colon-Ramos, D.A., Margeta, M.A., and Shen, K. (2007). Glia promote local synaptogenesis through UNC-6 (netrin) signaling in C. elegans. Science *318*, 103-106.

Dickinson, D.J., Pani, A.M., Heppert, J.K., Higgins, C.D., and Goldstein, B. (2015). Streamlined Genome Engineering with a Self-Excising Drug Selection Cassette. Genetics *200*, 1035-1049.

Ihara, S., Hagedorn, E.J., Morrissey, M.A., Chi, Q., Motegi, F., Kramer, J.M., and Sherwood, D.R. (2011). Basement membrane sliding and targeted adhesion remodels tissue boundaries during uterine-vulval attachment in Caenorhabditis elegans. Nat Cell Biol *13*, 641-651.

McMiller, T.L., and Johnson, C.M. (2005). Molecular characterization of HLH-17, a C. elegans bHLH protein required for normal larval development. Gene *356*, 1-10.

Nishiwaki, K., Hisamoto, N., and Matsumoto, K. (2000). A metalloprotease disintegrin that controls cell migration in Caenorhabditis elegans. Science *288*, 2205-2208.

Nonet, M.L., Staunton, J.E., Kilgard, M.P., Fergestad, T., Hartwieg, E., Horvitz, H.R., Jorgensen, E.M., and Meyer, B.J. (1997). Caenorhabditis elegans rab-3 mutant synapses exhibit impaired function and are partially depleted of vesicles. J Neurosci *17*, 8061-8073.

Okkema, P.G., Harrison, S.W., Plunger, V., Aryana, A., and Fire, A. (1993). Sequence requirements for myosin gene expression and regulation in Caenorhabditis elegans. Genetics *135*, 385-404.

Wenick, A.S., and Hobert, O. (2004). Genomic cis-regulatory architecture and trans-acting regulators of a single interneuron-specific gene battery in C. elegans. Dev Cell *6*, 757-770.
