# Supplementary material for: A muscle-epidermis-glia signaling axis sustains synaptic specificity during allometric growth in Caenorhabditis elegans
Source: eLife. 2020 Apr 7;9:e55890. doi: 10.7554/eLife.55890 (PMC7164957; doi:10.7554/eLife.55890)
Supplement: Transparent reporting form [file elife-55890-transrepform.docx]

***eLife’s* transparent reporting form**

We encourage authors to provide detailed information *within their submission* to facilitate the interpretation and replication of experiments. Authors can upload supporting documentation to indicate the use of appropriate reporting guidelines for health-related research (see [EQUATOR Network](http://www.equator-network.org/%20)), life science research (see the [BioSharing Information Resource](https://biosharing.org/)), or the [ARRIVE guidelines](http://www.plosbiology.org/article/info:doi/10.1371/journal.pbio.1000412) for reporting work involving animal research. Where applicable, authors should refer to any relevant reporting standards documents in this form.

If you have any questions, please consult our Journal Policies and/or contact us: [editorial@elifesciences.org](mailto:editorial@elifesciences.org).

**Sample-size estimation**

- You should state whether an appropriate sample size was computed when the study was being designed
- You should state the statistical method of sample size computation and any required assumptions
- If no explicit power analysis was used, you should describe how you decided what sample (replicate) size (number) to use

Please outline where this information can be found within the submission (e.g., sections or figure legends), or explain why this information doesn’t apply to your submission:

The sample sizes used in the study are indicated in the figure legends, in the figure graphs or both. The phenotypes examined have large effect sizes, as determined by blind scoring by multiple researchers, the fact that they were first identified from forwards genetic screens, and their use for positional mapping of the genetic lesion identified in the study. Sample sizes for scoring and statistical analyses were in the tens to hundreds of animals per phenotype. The sample size we chose is an over-kill for the power calculations, but we selected the sample size to capture the richness of the examined phenotypes in the populations of C. elegans worms and because of the practical ease with which substantial number of C. elegans can be examined, and the benefits those observations convey to the study.

As indicated in methods, scoring was done blind to genotype, and by multiple independent researchers to confirm and compare observations. Scoring was done across different days, and by examining/noting developmental stages, to control for variables that might affect the robust phenotypes observed.

**Replicates**

- You should report how often each experiment was performed
- You should include a definition of biological versus technical replication
- The data obtained should be provided and sufficient information should be provided to indicate the number of independent biological and/or technical replicates
- If you encountered any outliers, you should describe how these were handled
- Criteria for exclusion/inclusion of data should be clearly stated
- High-throughput sequence data should be uploaded before submission, with a private link for reviewers provided (these are available from both GEO and ArrayExpress)

Please outline where this information can be found within the submission (e.g., sections or figure legends), or explain why this information doesn’t apply to your submission:

All transgenic line markers were vetted by comparing the phenotypes in wild types with known electron microscopy (EM) connectomic studies and other controls, as indicated in methods. All markers and genotypes have been cryogenically stored, thawed and the reported phenotypes confirmed, so we have both the reproducible results, and the strains for confirmation of the observations by other labs. All experiments were performed over several days, looking at multiple generations of the same strains by multiple researchers in the study, for consensus/robustness/reproducibility of the observed phenotypes. The same marker lines were used across genotypes for standardized comparisons. When scoring rescue lines, at least three independent transgenic lines were scored, as indicated in the study. All the phenotypic data in the study is from biological replicates, the only technical replicates in the study are the ones performed for the LC/MS-MS studies, as indicated in the Methods. No outliers were excluded in the study.

**Statistical reporting**

- Statistical analysis methods should be described and justified
- Raw data should be presented in figures whenever informative to do so (typically when N per group is less than 10)
- For each experiment, you should identify the statistical tests used, exact values of N, definitions of center, methods of multiple test correction, and dispersion and precision measures (e.g., mean, median, SD, SEM, confidence intervals; and, for the major substantive results, a measure of effect size (e.g., Pearson's r, Cohen's d)
- Report exact p-values wherever possible alongside the summary statistics and 95% confidence intervals. These should be reported for all key questions and not only when the p-value is less than 0.05.

Please outline where this information can be found within the submission (e.g., sections or figure legends), or explain why this information doesn’t apply to your submission:

Specified statistical analyses were based on student’s t-test for comparisons between two groups or one-way ANOVA by Tukey’s multiple comparison test for three or more groups. All statistical reporting is provided in the figure legends and methods section.

(For large datasets, or papers with a very large number of statistical tests, you may upload a single table file with tests, Ns, etc., with reference to sections in the manuscript.)

**Group allocation**

- Indicate how samples were allocated into experimental groups (in the case of clinical studies, please specify allocation to treatment method); if randomization was used, please also state if restricted randomization was applied
- Indicate if masking was used during group allocation, data collection and/or data analysis

Please outline where this information can be found within the submission (e.g., sections or figure legends), or explain why this information doesn’t apply to your submission:

*C. elegans* samples were divided by the indicated genotypes, which were all confirmed by genotyping, or by the stage of the animal, which was confirmed based on standard anatomical features or synchronized rearing, as indicated in the methods section.

**Additional data files (“source data”)**

- We encourage you to upload relevant additional data files, such as numerical data that are represented as a graph in a figure, or as a summary table
- Where provided, these should be in the most useful format, and they can be uploaded as “Source data” files linked to a main figure or table
- Include model definition files including the full list of parameters used
- Include code used for data analysis (e.g., R, MatLab)
- Avoid stating that data files are “available upon request”

Please indicate the figures or tables for which source data files have been provided:

Table S1 lists protein fold changes from proteomic analyses of *mig-17(ola226)* mutants as compared to wild type animals.
